# Cost-effectiveness of HIV prevention interventions in Andhra Pradesh state of India

**DOI:** 10.1186/1472-6963-10-117

**Published:** 2010-05-10

**Authors:** Lalit Dandona, SG Prem Kumar, G Anil Kumar, Rakhi Dandona

**Affiliations:** 1 Public Health Foundation of India, ISID Campus, 4 Institutional Area, Vasant Kunj, New Delhi 110070, India; 2 Institute for Health Metrics and Evaluation, University of Washington, 2301 5th Avenue, Seattle, WA 98121, USA

## Abstract

**Background:**

Information on cost-effectiveness of the range of HIV prevention interventions is a useful contributor to decisions on the best use of resources to prevent HIV. We conducted this assessment for the state of Andhra Pradesh that has the highest HIV burden in India.

**Methods:**

Based on data from a representative sample of 128 public-funded HIV prevention programs of 14 types in Andhra Pradesh, we have recently reported the number of HIV infections averted by each type of HIV prevention intervention and their cost. Using estimates of the age of onset of HIV infection, we used standard methods to calculate the cost per Disability Adjusted Life Year (DALY) saved as a measure of cost-effectiveness of each type of HIV prevention intervention.

**Results:**

The point estimates of the cost per DALY saved were less than US $50 for blood banks, men who have sex with men programmes, voluntary counselling and testing centres, prevention of parent to child transmission clinics, sexually transmitted infection clinics, and women sex worker programmes; between US $50 and 100 for truckers and migrant labourer programmes; more than US $100 and up to US $410 for composite, street children, condom promotion, prisoners and workplace programmes and mass media campaign for the general public. The uncertainty range around these estimates was very wide for several interventions, with the ratio of the high to the low estimates infinite for five interventions.

**Conclusions:**

The point estimates for the cost per DALY saved from the averted HIV infections for all interventions was much lower than the per capita gross domestic product in this Indian state. While these indicative cost-effectiveness estimates can inform HIV control planning currently, the wide uncertainty range around estimates for several interventions suggest the need for more firm data for estimating cost-effectiveness of HIV prevention interventions in India.

## Background

India has had substantial resources allocated for HIV/AIDS control of which the major portion would be spent on HIV prevention [1,2]. Efficient use of these resources would take into account the gaps in coverage of HIV prevention interventions, the cost of interventions and their cost-effectiveness. The state of Andhra Pradesh in south India, with a population of over 80 million, is estimated to have the highest burden of HIV among all states in India [3]. We have recently reported an economic analysis of the entire range of public-funded HIV prevention interventions in Andhra Pradesh, taking into account their cost, the estimated HIV infections that could be prevented by these interventions, and the gaps in their coverage [4]. We now report estimates of the cost-effectiveness of these HIV prevention interventions and discuss the issues involved in these estimations.

## Methods

### Sample and data collection

The Andhra Pradesh State AIDS Control Society (APSACS) is the nodal agency that facilitates a variety of public-funded HIV prevention interventions. Four of these interventions are based in clinical settings: voluntary counselling and testing (VCT) centres, prevention of parent to child transmission (PPTCT) centres, sexually transmitted infection (STI) clinics, and blood banks. VCT and PPTCT are now offered at the same facility referred to as integrated counselling and testing (ICT) centre. Eight interventions are implemented by non-governmental organisations targeting groups at high-risk of HIV: women sex worker (SW) programmes, men who have sex with men (MSM) programmes, truckers programmes, composite programmes, workplace programmes, migrant labourer programmes, street children programmes, and prisoner programmes. In addition, APSACS implements a state-wide information, education and communication (IEC) programme for the general public (mass media campaigns), and previously used to implement the state-wide condom promotion programme. Of the total 551 programmes facilitated by APSACS during the April 2005 to March 2006 fiscal year, a representative sample of 128 programmes was selected (Table 1) [4]. Data on the HIV prevention services provided by these programmes, and the economic cost of the programmes including personnel, recurrent goods, recurrent services, capital goods and office space rentals, were documented from written records and interviews of programme staff by trained investigators using procedures described previous that included quality control [4-10]. The adolescent education awareness programme for HIV prevention that is facilitated by the State Council of Education Research and Training was not included in this assessment.

**Table 1:** Sampled programmes from among the public-funded HIV prevention interventions facilitated by APSACS in Andhra Pradesh in the 2005-2006 fiscal year [4].

Interventions	Total number of programmes	Number of programmes sampled*
VCT centres	198	25

STI clinics	85	22

PPTCT clinics	101	16

Blood banks	56	14

Women SW programmes	20	16

MSM programmes	3	2

Trucker programmes	21	6

Composite programmes	23	6

Workplace programmes	4	3

Migrant labourer programmes	7	4

Street children programmes	6	3

Prisoner programmes	25	9

IEC for general public	1	1

Condom promotion programme	1	1

**Total**	**551**	**128**

*Sample of programmes selected for each intervention using stratification by the three regions of the state, and in some cases stratification also by sub-type of intervention when applicable, to obtain representation proportional to the actual distribution.

### Cost-effectiveness calculations

We have previously reported the total HIV prevention services provided through APSACS facilitated programs and the economic cost of these services in Andhra Pradesh in the 2005-2006 fiscal year, and based on the estimated intervention effect per unit service we have reported the number of HIV infections averted by each intervention in this year along with the uncertainty range calculated from sensitivity analysis with Monte Carlo simulations using random values of input variables (Table 2) [4]. This method and the detailed calculations of HIV infections averted by each intervention are shown in Additional file 1 as compared with the situation if there was no intervention at all. From this we calculated the cost per HIV infection averted during the 2005-2006 fiscal year by each type of prevention intervention for the services provided in Andhra Pradesh during this year. We then calculated Disability Adjusted Life Years (DALYs) saved as the sum of the Years of Life Lost (YLL) and the Years Lost due to Disability (YLD) that would be saved for each HIV infection averted by each prevention intervention using published methods [11-14].

**Table 2:** Services provided by public-funded HIV prevention interventions in Andhra Pradesh, their economic cost and the estimated HIV cases averted in the 2005-2006 fiscal year [4].

Intervention	Number of persons served*	Total programme cost INR (US$)†	Estimated number of HIV infections averted‡	
			
			Point estimate	Uncertainty range
VCT centres	261,421	38,552,074 (870,840)	1,310	327 - 2,800

PPTCT clinics	217,274	21,496,098 (485,568)	592	463 - 747

STI clinics	116,735	84,656,539 (1,912, 278)	2,315	1,705 - 3,064

Blood banks	107,912	7,570,027 (170,997)	1,755	1,431 - 2,082

Women SW programmes	40,732	57,915,975 (1,308,244)	1,330	629 - 2,164

MSM programmes	18,270	6,268,799 (141,604)	611	295 - 930

Trucker programmes	494,354	54,529,010 (1,231,737)	681	0 - 1,256

Composite programmes	147,194	39,122,674 (883,729)	250	65 - 417

Workplace programmes	19,855	7,713,881 (174,246)	22	7 - 35

Migrant labourer programmes	113,356	19,337,166 (436,801)	202	76 - 320

Street children programmes	46,892	8,802,798 (198,843)	38	0 - 130

Prisoner programmes	146,599	16,358,548 (369,518)	46	0 - 211

IEC for general public	33,000,000	234,846,584 (5,304,870)	498	0 - 4,290

Condom promotion programme	190,599	11,648,524 (263,125)	39	0 - 128

*As reported for the public-funded programmes in the 2005-2006 fiscal year; for blood banks the number is the blood units screened; IEC estimate based on coverage of the 15-49 years old age group in Andhra Pradesh with HIV mass media campaigns through television, radio, print media and public events.

†INR is India Rupee; 1 US$ = INR 44.27 in the 2005-2006 fiscal year [15].

‡Point estimate calculated from HIV infections averted per unit service multiplied by the total number of persons served; uncertainty range calculated from the 5^th ^and 95^th ^percentile values for HIV infections averted per unit service obtained from 100,000 Monte Carlo simulations with @Risk software using random values of input variables [4].

Where a = age at death in years; L = standard expectation of life in years at age a; r = discount rate; K = age-weighting modulation factor; C = age-weighting adjustment constant; β = constant from the age-weighting function.

Where a = age at onset of disability in years; L = duration of disability in years; D = disability weight; r = discount rate; K = age-weighting modulation factor; C = age-weighting adjustment constant; β = constant from the age-weighting function.

We used the commonly used discount rate r = 0.03, i.e. 3% yearly discount rate for the value of years of life lost in the future. For calculation of DALYs with age-weighting, previously suggested values for constants were used: K = 1, C = 0.1658 and β = 0.04. As the use of age-weighting in the calculation of DALYs is controversial due to preferential weights given to young adults as compared with children and elderly, we calculated DALYs without age-weighting in which the value of the age-weighting modulation factor K = 0 and C and β become not applicable.

Based on estimated local trends, for sexually acquired HIV in adults the average age at infection was assumed as 30 years for men and 25 years for women. For sexually acquired HIV in street children, the average age at infection was assumed as 17 years for both boys and girls based on estimated local trends. The age at infection for mother to child transmission of HIV was considered as the first year of life. The average age at infection from blood transfusion was estimated as 5 years in the 14 years and less age group, 30 years in the 15-44 years age group, and 50 years in the 45 years and above age group, based on local trends. DALYs were calculated separately for men and women and a weighted average calculated based on the proportion of HIV infections averted in men and women by each intervention.

The YLDs were calculated separately for pre-AIDS and AIDS. The duration of HIV to pre-AIDS was considered as 6 years, from pre-AIDS to AIDS as 2 years, and from AIDS to death as 1.5 years without anti-retroviral treatment [15]. As a very small fraction of persons with HIV/AIDS were on antiretroviral treatment in India at the time of this study, we calculated DALYs saved without including prolonged life and additional cost due to antiretroviral treatment. This would have to be re-considered as the antiretroviral treatment becomes more common.

DALYs saved for each HIV infection averted by each intervention were computed with and without age-weighting as YLLs + YLDs (Pre AIDS) + YLDs (AIDS). Utilising the cost per HIV infection averted computations, we then calculated the cost per DALY saved for each intervention. Cost in Indian Rupees (INR) was converted to US$ using the average exchange rate of INR 44.27 for a US$ in the 2005-2006 fiscal year [16]. We calculated the uncertainty range of these estimates based on the 5^th ^and 95^th ^percentile values of HIV infections averted by each intervention computed previously with sensitivity analysis through 100,000 iterations each by Monte Carlo simulations using the @Risk software (Palisade Corporation, Newfield, New York, USA).

## Results

The lowest cost per HIV infection averted was that by blood banks (INR 4,313, US $97) followed by MSM programmes (INR 10,265, US $232) and VCT clinics, PPTCT clinics, STI clinics and women sex worker programmes (INR 29,425-43,554, US $665-984) (Table 3). Truckers programmes cost INR 80,116 (US $1,810) and migrant labourer programmes cost INR 95,943 (US $2,167) per HIV infection averted, with the other programmes having a cost of INR 156,520-471,676 (US $3,536-10,655) per HIV infection averted.

**Table 3:** Cost per HIV infection averted for public-funded HIV prevention interventions in Andhra Pradesh state of India.

Intervention	Cost per HIV infection averted* INR (US$)	
	
	Point estimate	Uncertainty range
VCT centres	29,425 (665)	13,769 - 117,977 (311 - 2,665)

PPTCT clinics	36,329 (821)	28,760 - 46,449 (650 - 1,049)

STI clinics	36,568 (826)	27, 627 - 49, 637 (624 - 1,121)

Blood banks	4,313 (97)	3,637 - 5,290 (82 - 120)

Women SW programmes	43,554 (984)	26,767 - 92,031 (605 - 2,079)

MSM programmes	10,265 (232)	6,744 - 21,246 (152 - 480)

Trucker programmes	80,116 (1,810)	43,427 - infinite (981 - infinite)

Composite programmes	156,520 (3,536)	93,919 - 604,068 (2,121 - 13,645)

Workplace programmes	357,057 (8,065)	223,282 - 1,110,031 (5,044 - 25,074)

Migrant labourer programmes	95,943 (2,167)	60,492 - 254,609 (1,366 - 5,751)

Street children programmes	229,524 (5,185)	67,771 - infinite (1,531 - infinite)

Prisoner programmes	353,576 (7,987)	77,491 - infinite (1,750 - infinite)

IEC for general public	471,676 (10,655)	54,743 - infinite (1,237 - infinite)

Condom promotion programme	299,823 (6,773)	91,217 - infinite (2,060 - infinite)

*Calculated from programme cost and number of HIV infections averted in Table 2; figures presented to the nearest INR and nearest US$.

The DALYs saved per HIV infection averted were somewhat lower without age-weighting than with age-weighting the calculations for which are shown in the Additional file 2, and therefore the cost per DALY saved was somewhat higher without age-weighting (Tables 4 &5). Blood banks and MSM programmes had the lowest cost per DALY saved, INR 176 (US $4) and INR 414 (US $9) respectively without age-weighting (Table 5). The cost per DALY saved without age weighting for VCT clinics, PPTCT clinics, STI clinics and women sex worker programmes was INR 1,119-1,662 (US $25-38). This was followed by INR 6,043-8,316 (US $137-188) per DALY saved for composite and street children programmes, and INR 11,532-18,141 (US $260-410) for composite, prisoners, workplace and IEC programmes (Table 5).

**Table 4:** Cost per DALY saved with age-weighting for public-funded HIV prevention interventions in Andhra Pradesh state of India.

Intervention	DALYs saved per HIV infection averted* (with age-weighting)	Cost per DALY saved† (with age-weighting) INR (US$)	
		
		Point estimate	Uncertainty range
VCT centres	28.1	1,047 (24)	490 - 4,198 (11 - 95)

PPTCT clinics	38.4	946 (21)	749 - 1,210 (17 - 27)

STI clinics	28.0	1,306 (30)	987 - 1,773 (22 - 40)

Blood banks	25.4	170 (4)	143 - 208 (3 - 5)

Women SW programmes	27.8	1,567 (35)	963 - 3,310 (22 - 75)

MSM programmes	25.3	406 (9)	267 - 840 (6 - 19)

Trucker programmes	27.4	2,924 (66)	1,585 - infinite (36 - infinite)

Composite programmes	27.3	5,733 (130)	3,440 - 22,127 (78 - 500)

Workplace programmes	27.4	13,031 (294)	8,149 - 40,512 (184 - 915)

Migrant labourer programmes	27.4	3,502 (79)	2,208 - 9,292 (50 - 210)

Street children programmes	33.1	6,934 (157)	2,047 - infinite (46 - infinite)

Prisoner programmes	27.4	12,904 (291)	2,828 - infinite (64 - infinite)

IEC for general public	27.4	17,214 (389)	1,998 - infinite (45 - infinite)

Condom promotion programme	27.4	10,942 (247)	3,329 - infinite (75 - infinite)

*Calculations for DALYs saved per HIV infection averted by each intervention shown in the Additional file.

†Calculated from cost per HIV infection averted in Table 3 and DALYs saved per HIV infection; figures presented to the nearest INR and nearest US$.

**Table 5:** Cost per DALY saved without age-weighting for public-funded HIV prevention interventions in Andhra Pradesh state of India.

Intervention	DALYs saved per HIV infection averted* (without age-weighting)	Cost per DALY saved† (without age-weighting) INR (US$)		Ratio of high to low estimates in uncertainty range
			
		Point estimate	Uncertainty range	
VCT centres	26.3	1,119 (25)	524 - 4,486 (12 - 101)	8.6

PPTCT clinics	29.9	1,215 (27)	962 - 1,553 (22 - 35)	1.6

STI clinics	26.3	1,390 (31)	1,050 - 1,887 (24 - 43)	1.8

Blood banks	24.5	176 (4)	148 - 216 (3 - 5)	1.5

Women SW programmes	26.2	1,662 (38)	1,022 - 3,513 (23 - 79)	3.4

MSM programmes	24.8	414 (9)	272 - 857 (6 - 19)	3.2

Trucker programmes	26.0	3,081 (70)	1,670 - infinite (38 - infinite)	infinite

Composite programmes	25.9	6,043 (137)	3,626 - 23,323 (82 - 527)	6.4

Workplace programmes	26.0	13,733 (310)	8,588 - 42,693 (194 - 964)	5

Migrant labourer programmes	25.9	3,704 (84)	2,336 - 9,830 (53 - 222)	4.2

Street children programmes	27.6	8,316 (188)	2,455 - infinite (55 - infinite)	infinite

Prisoner programmes	26.0	13,599 (307)	2,980 - infinite (67 - infinite)	infinite

IEC for general public	26.0	18,141 (410)	2,105 - infinite (48 - infinite)	infinite

Condom promotion programme	26.0	11,532 (260)	3,508 - infinite (79 - infinite)	infinite

*Calculations for DALYs saved per HIV infection averted by each intervention shown in the Additional file.

†Calculated from cost per HIV infection averted in Table 3 and DALYs saved per HIV infection; figures presented to the nearest INR and nearest US$.

The ratio of the highest to the lowest value in the cost-effectiveness uncertainty range was less than 2 for blood banks, PPTCT and STI clinics, 3-5 for MSM, women SW, migrant labourer and workplace programmes, 6-9 for composite programmes and VCT clinics, and infinite for truckers, street children, prisoners, IEC and condom promotion programmes based on sensitivity analysis using the plausible range of input variables (Table 5).

## Discussion

Cost-effectiveness estimates for HIV prevention interventions are scarce in India. This is a useful component of the evidence base needed for efficient use of available resources to prevent HIV [17]. When resources are scarce, cost-effectiveness estimates help make choices where to put the money for maximum health benefit. However, decisions on resource allocation also take into account many factors other than cost-effectiveness. Our estimates of the cost-effectiveness of the range of public-funded HIV prevention interventions in Andhra Pradesh state reported in this paper are the most comprehensive information available on this topic so far from India.

The World Bank a decade ago as part of its project appraisal for the second national HIV/AIDS control programme of India estimated the cost per DALY saved as US$2.7 for sex worker programmes, US$2.4 for STI management and US$10 for VCT [18]. In another previous report based on data from a sex worker programme during 1999-2003 in Gujarat, the cost per DALY saved was reported as US$5.5 with an uncertainty range of US$3-12 [19]. There are substantial differences between these estimates and ours reported in this paper. The cost per DALY saved estimates for sex worker programmes by the World Bank and the Gujarat study, and for STI management by the World Bank, are an order of magnitude lower than our estimates of US$38 (uncertainty range US$23-79) for sex worker programmes and US$31 (uncertainty range US$24-43) for STI clinics. The cost per DALY saved estimate for VCT by the World Bank is 2.5 times lower than our estimate of US$25 (uncertainty range US$12-101). The previous lower point estimates for the cost per DALY saved are likely due to different assumptions used in the HIV transmission models. We have previously reported the basis of the validity of our transmission model on which our cost-effectiveness assumptions are based [4]. The large differences between our cost-effectiveness estimates and those reported previously for sex worker programmes, STI management and VCT merit further probing with actual longitudinal data to estimate the impact of these interventions in preventing HIV.

The World Bank project appraisal for the second national HIV/AIDS control programme also estimated a cost of US$128 per DALY saved for mother to child transmission intervention with ziduvidine (AZT) [18]. Our estimate of US$27 (uncertainty range US$22-35) per DALY saved for PPTCT clinics with nevirapine is about five times lower than the previous World Bank estimate a major reason for which is the much lower cost of nevirapine now than that of AZT at the time of World Bank estimates a decade ago. Another economic analysis has estimated a cost of $586 per HIV infection averted for nationwide screening of pregnant women and treatment with nevirapine in India and $275 per HIV infection averted if this programme were implement only in high HIV prevalence states including Andhra Pradesh [20]. Our estimate of US$821 (uncertainty range US$650-1049) per HIV infection averted by PPTCT clinics is about three times higher than the previous estimate for high prevalence states. While this may be partly due to the previous higher HIV prevalence estimates in the general population and pregnant women in India, which have dropped down by about half due to the availability of recent population-based data [21,22], this may be due to other methodological differences too. This again underscores the need for actual longitudinal data to estimate the HIV infections averted and cost-effectiveness.

As compared with the per capita gross domestic product (GDP) of Andhra Pradesh state (INR 28,063, US $634) [23], the point estimates of the cost per DALY saved for six HIV prevention interventions in Andhra Pradesh were less than 10% of the per capita GDP, all except one intervention were less than half the per capita GDP, and for one intervention was 65% of the per capita GDP, indicating that these interventions were generally quite cost-effective. Interventions costing less than per capita GDP are considered highly cost-effective [24]. However, a striking finding of our analysis is the very wide uncertainty range of the cost-effectiveness estimates for several HIV prevention interventions. The infinite ratio of the highest to the lowest values in the uncertainty range for truckers, street children, prisoners, IEC and condom promotion programmes, and a ratio of 9 for VCT, suggest that more concrete data are required for input variables that go into the estimation of HIV infection averted by these interventions which in turn directly affect the cost-effectiveness estimates. We therefore suggest that the cost-effectiveness estimates reported by us that have a very wide uncertainty ranges should be considered only indicative at this stage. On the other hand, the upper end of uncertainty range of the cost per DALY saved was less than half the per capita GDP for blood banks, PPTCT clinics, STI clinics, VCT centres, MSM programmes, women sex worker programmes and migrant labourer programmes, suggesting that even at the high end of uncertainty range of the current estimates these HIV prevention interventions have good cost-effectiveness.

There are some limitations of our analysis. Both the cost of interventions and the calculation of HIV infections averted that we computed for cost-effectiveness estimates are for a single year. While the intervention effect may also have a beneficial impact on preventing HIV in subsequent years, we do not have adequate data on which to base justifiable assumptions and calculations for this. Not including this aspect would tend to underestimate the cost-effectiveness of interventions in our calculations. However, as we present similar cross-sectional annual calculations for each intervention, our approach is consistent across interventions. Neither the future treatment cost of HIV/AIDS and associated diseases averted by prevention of HIV infection, nor the DALYs that would be saved if that treatment cost were incurred, are included in our cross-sectional calculations. Increasingly common availability of anti-retroviral treatment in India could influence the cost-effectiveness calculations if the cost of treatment per additional DALY saved were substantially higher or lower than the cost per DALY saved due to HIV prevention interventions. We are unable to comment which way this would go, but for now our cross-sectional single year calculations are consistent for all interventions.

The trends of cost-effectiveness of the various public-funded HIV prevention interventions in our analysis from Andhra Pradesh state may be applicable to the other high HIV burden states in southern India, but it would be difficult to generalise these trends to the other states which have a lower level of HIV burden and less developed HIV prevention services at this stage. In addition to the public-funded HIV prevention interventions analysed in this paper, it would be useful to understand the cost-effectiveness of HIV prevention interventions funded from other sources. In this regard, the cost-effectiveness assessment being undertaken as part of the evaluation of the Gates Foundation funded Avahan AIDS India Initiative is likely to provide useful complementary data [25].

It should also be noted that cost-effectiveness estimates would evolve as the HIV interventions are scaled up. We have previously reported that the unit cost of services for sex workers doubled over three years in Andhra Pradesh due to increase in the intensity of services suggestive of better quality services, and the unit cost of VCT reduced to half over this period due to higher client volume without an indication of decrease in quality of services [9]. The increase in scale of services at some stage may also result in less efficiency as more effort has to be spent in order to get to the resistant hard to reach sub-groups. Therefore several factors come into play that influence the cost-effectiveness in opposite directions at various stages of the development of HIV prevention interventions. Understanding the evolution of cost-effectiveness would benefit from actual longitudinal data on HIV infections averted by the prevention interventions in India.

## Conclusions

The cost-effectiveness estimates of the range of public-funded HIV prevention interventions reported in this paper are a useful addition to the evidence base needed for an efficient use of resources to control HIV in India. The point estimates for the cost per DALY saved due to the averted HIV infections was much lower than the per capita gross domestic product for all interventions. Major differences between these estimates of cost-effectiveness and some reported by others earlier underscore the need for standardized and explicit methodology for such estimates. The very wide uncertainty range for several of our cost-effectiveness estimates highlights the need for more robust actual longitudinal data in India for more firm estimates.

## Competing interests

The authors declare that they have no competing interests.

## Authors' contributions

LD conceived this study, led the design, analysis and interpretation, and drafted the manuscript. SGPK, GAK and RD contributed to the design, analysis, and interpretation. All authors approved the final version of the manuscript.

## Pre-publication history

The pre-publication history for this paper can be accessed here:

http://www.biomedcentral.com/1472-6963/10/117/prepub

## Supplementary Material

Additional file 1 **Calculation of the effect of interventions on reducing HIV**. This file shows calculation of HIV infections averted by each HIV prevention intervention in Andhra Pradesh state of India. Click here for file

Additional file 2 **Calculation of Disability Adjusted Life Years (DALYs) saved**. This file shows calculation of Disability Adjusted Life Years (DALYs) saved for each HIV infection averted by HIV prevention interventions in Andhra Pradesh state of India. Click here for file
