# Supplementary material for: Cost-effectiveness of HIV prevention interventions in Andhra Pradesh state of India
Source: BMC Health Serv Res. 2010 May 10;10:117. doi: 10.1186/1472-6963-10-117 (PMC2874552; doi:10.1186/1472-6963-10-117)
Supplement: Additional file 1 — Calculation of the effect of interventions on reducing HIV. This file shows calculation of HIV infections averted by each HIV prevention intervention in Andhra Pradesh state of India. [file 1472-6963-10-117-S1.DOC]

**Cost-effectiveness of HIV prevention interventions in Andhra Pradesh state of India**

Lalit Dandona, S G Prem Kumar, G Anil Kumar, Rakhi Dandona

**Additional file 1:** Calculation of the effect of interventions on reducing HIV.

This file shows calculation of HIV infections averted by each HIV prevention intervention in Andhra Pradesh state of India.

We have previously published the method used to calculate the number of HIV infections averted by each HIV prevention intervention.^1^ A description of this method is provided here followed by detailed calculations for HIV infections averted by each intervention starting on page 4.

The majority of interventions target sex behaviour for which the base scenario of the probability of HIV infection without intervention in a group that would be the beneficiary of the intervention was estimated using the Weinstein formula that is used widely including by UNAIDS^2,3^ which is:

Pr = 1-{P[1-R(1-FE)]^N^+(1-P)}^M^

where Pr is the probability of HIV infection in uninfected, P is the average HIV prevalence among sex partners of the group for which probability is being estimated, R is the risk of HIV acquisition per act of unprotected sex, F is the fraction of sex acts in which condom is used, E is the effectiveness of condoms, N is the average number of sex acts per partner, and M is the average number of sex partners. In order to estimate the probability of new HIV infection in an entire group, Pr was multiplied by (1 – I), where I is the proportion in the susceptible group that is already infected with HIV. Probabilities were calculated separately for acquisition of HIV by the group that would receive the prevention intervention and for their sex partners, and the number of new infections from these added to obtain the total new HIV infections. For women SW intervention this also included acquisition of HIV by the other women sex partners of clients of sex workers, and for MSM intervention this included acquisition of HIV by women sex partners of MSM.

The probabilities of HIV acquisition per act of unprotected sex were adapted from the literature.^3-6^ For groups at high risk of HIV, in the absence of STI we used a probability of 0.0014 for receptive vaginal sex, 0.0007 for insertive vaginal sex, 0.01 for receptive anal sex, 0.001 for insertive anal sex, and 0.0004 for receptive oral sex. These probabilities were considered three times higher in the presence of STI.^3,6^ For IEC intervention for the general public, 30% lower probabilities of HIV acquisition per act of unprotected sex were used, assuming less risky sex as compared with other groups at higher risk.^6^ The effectiveness of condom in reducing HIV transmission in vaginal sex was taken as 80%, in anal sex as 70% and in oral sex as 90%.^7,8^ The values for HIV prevalence, fraction of sex acts in which condom was used, average number of sex acts per partner and the average number of sex partners were adapted from population-based and other surveys^8-14^ and programme data.

To assess the effect of interventions in reducing HIV in the groups that they serve, the estimated impact of interventions on reducing condom non-use, number of partners and STI non-treatment from a recent global report on low- and middle-income countries were adapted for use – we mostly used medium values from this source.^15,16^ For impact values not available from this source for certain interventions, estimates were adapted from comparable interventions using assumptions informed by understanding of local trends in Andhra Pradesh. Based on these most plausible impact values, the Weinstein formula was applied to the with-intervention situation, with changed values for condom use, number of partners and risk of HIV transmission per act of unprotected sex (due to impact on STI treatment) for an estimate of the number of HIV cases averted per 1000 persons receiving an intervention. These estimates took into account the effect of overlapping target groups by different interventions and of overlapping sex partners within an intervention.

The base scenario and intervention effect for PPTCT were based on data on pregnant women receiving PPTCT and the proportion of HIV positive receiving nevirapine, assuming an HIV transmission rate without treatment as 25% and a reduction in this transmission by 40% with nevirapine treatment.^17^ The base scenario and intervention effect for blood banks were based on the proportion of blood units that tested HIV positive and an estimated 92% risk of HIV transmission from infected blood.^18^

Published literature, unpublished population-based and programme data, and assumptions based on our understanding of local trends were used to estimate the range of plausible values for each variable (listed later in this document starting on page 41). Using random values between these plausible ranges, sensitivity analyses for the intervention effects were performed based on the Monte Carlo simulation principle with 100,000 iterations using the @Risk software (Palisade Corporation, Newfield, New York, USA) to obtain the 5^th^ and 95^th^ percentile values of the number of HIV infections averted by each intervention.

**References**

1. Dandona L, Kumar SGP, Kumar GA, Dandona R. Economic analysis of HIV prevention interventions in Andhra Pradesh state of India to inform resource allocation. *AIDS* 2009; 23: 233-242.
2. Weinstein M, Graham J, Siegel J, Fineberg H. Cost-effectiveness analysis of AIDS prevention programs: concepts, complications and illustrations. In: Turner C, Miller H, Moses L (editors). AIDS: *Sexual Behavior and Intravenous Drug Use*. Washington, DC: National Academy Press, 1989.
3. Joint United Nations Programme on HIV/AIDS. *Epidemiological software and tools (2007):* ***modes of transmission spreadsheets and manuals*. Available at: http://www.unaids.org/en/KnowledgeCentre/HIVData/Epidemiology/epi_software2007.asp.**
4. Royce RA, Sena A, Cates W Jr, Cohen MS. Sexual transmission of HIV. *N Engl J Med* 1997; 336: 1072-1078.
5. Vittinghoff E, Douglas J, Judson F, McKirnan D, MacQueen K, Buchbinder SP. Per-contact risk of human immunodeficiency virus transmission between male sexual partners. *Am J Epidemiol* 1999; 150: 306-311.
6. Gouws E, White PJ, Stover J, Brown T. Short term estimates of adult HIV incidence by mode of transmission: Kenya and Thailand as examples. *Sex Transm Infect* 2006; 82(Suppl 3): iii51-55.
7. Weller S, Davis K. Condom effectiveness in reducing heterosexual HIV transmission. *Cochrane Database Syst Rev* 2002; 1: CD003255.
8. Dandona L, Dandona R, Kumar GA, Gutierrez JP, McPherson S, Bertozzi SM, et al. How much attention is needed towards men who sell sex to men for HIV prevention in India? *BMC Public Health* 2006; 6: 31.
9. Dandona L, Lakshmi V, Sudha T, Kumar GA, Dandona R. A population-based study of human immunodeficiency virus in south India reveals major differences from sentinel surveillance-based estimates. *BMC Med* 2006; 4: 31.
10. National AIDS Control Organization, Ministry of Health & Family Welfare, Government of India. *National behavioural surveillance survey 2006: general population*. Available at: http://www.nacoonline.org/upload/NACO%20PDF/General_Population.pdf.
11. National AIDS Control Organization, Ministry of Health & Family Welfare, Government of India. *National behavioural surveillance survey 2006: female sex workers and their clients*. Available at: http://www.nacoonline.org/upload/NACO%20PDF/General_Population.pdf.
12. Dandona R, Dandona L, Kumar GA, Gutierrez JP, McPherson S, Samuels F, et al. Utilising demography and sex work characteristics of female sex workers to enhance HIV prevention programmes in India. *BMC International Health and Human Rights* 2006; 6: 5.
13. Dandona L, Dandona R, Gutierrez JP, Kumar GA, McPherson S, Bertozzi SM, et al. Sex behaviour of men who have sex with men and risk of HIV in Andhra Pradesh, India. *AIDS* 2005; 19: 611-619.
14. Dandona L, Dandona R, Kumar GA, Reddy GB, Ameer MA, Ahmed GM, et al. Risk factors associated with HIV in a population-based study in Andhra Pradesh state of India. *Int J Epidemiol* 2008; 37: 1274-1286.
15. Stover J, Bertozzi S, Gutierrez JP, Walker N, Stanecki KA, Greener R, et al. The global impact of scaling up HIV/AIDS prevention programs in low- and middle-income countries. *Science* 2006; 311: 1474-1476.
16. Bollinger L, Cooper-Arnold C, Stover J. Where are the gaps? The effects of HIV-prevention interventions on behavioral change. *Stud Fam Plann* 2004; 35: 27-38.
17. World Health Organization. *Antiretroviral drugs for treating pregnant women and preventing HIV infection in infants*. Geneva: WHO, 2004.
18. Baggaley RF, Boily MC, White RG, Alary M. Risk of HIV-1 transmission for parenteral exposure and blood transfusion: a systematic review and meta-analysis. *AIDS* 2006; 20: 805-812.

**Intervention effectiveness calculations**

Calculations for the effectiveness of each intervention in averting new HIV infections, using the methods described above are as follows.

**Voluntary counselling and testing (VCT) centres**

**Men**

**Annual new HIV infections – base scenario without intervention**

|  |  | **Risk of acquiring HIV** | | | **Risk of transmitting HIV** | | |
| --- | --- | --- | --- | --- | --- | --- | --- |
|  |  | **Insertive vaginal** | **Insertive anal** | **Insertive oral** | **Receptive vaginal** | **Receptive anal** | **Receptive oral** |

| HIV prevalence in sex partners* | **P** | 0.08 | 0.08 | 0.08 | 0.20 | 0.20 | 0.20 |
| --- | --- | --- | --- | --- | --- | --- | --- |
| Risk of HIV per unprotected act† | **R** | 0.0010 | 0.0014 | 0.0000 | 0.0020 | 0.0140 | 0.0006 |
| Fraction of acts with condom‡ | **F** | 0.30 | 0.30 | 0.10 | 0.30 | 0.30 | 0.10 |
| Effectiveness of condom§ | **E** | 0.80 | 0.70 | 0.90 | 0.80 | 0.70 | 0.90 |
| Number of acts per partner¶ | **N** | 25 | 25 | 25 | 25 | 25 | 25 |
| Number of partners¶ | **M** | 4 | 0.004 | 0.01 | 4 | 0.004 | 0.01 |
| Probability |  | 0.00589 | 0.00001 | 0.00000 | 0.02895 | 0.00020 | 0.00003 |
| Total per 1000 |  | 5.89 | 0.01 | 0 | 28.95 | 0.20 | 0.03 |
| Proportion already infected | **I** | 0.20 | 0.20 | 0.20 | 0.08 | 0.08 | 0.08 |
| Total per 1000 - I adjusted |  | 4.71 | 0.01 | 0.00 | 26.63 | 0.18 | 0.02 |
|  |  |  |  | **4.7** |  |  | **26.8** |

**Annual new HIV infections with VCT intervention**

|  |  | **Risk of acquiring HIV** | | | **Risk of transmitting HIV** | | |
| --- | --- | --- | --- | --- | --- | --- | --- |
|  |  | **Insertive vaginal** | **Insertive anal** | **Insertive oral** | **Receptive vaginal** | **Receptive anal** | **Receptive oral** |
| HIV prevalence in sex partners | **P** | 0.08 | 0.08 | 0.08 | 0.20 | 0.20 | 0.20 |
| Risk of HIV per unprotected act | **R** | 0.0010 | 0.0014 | 0.0000 | 0.0020 | 0.0140 | 0.0006 |
| Fraction of acts with condom ׀׀ | **F** | 0.47 | 0.47 | 0.10 | 0.47 | 0.47 | 0.10 |
| Effectiveness of condom | **E** | 0.80 | 0.70 | 0.90 | 0.80 | 0.70 | 0.90 |
| Number of acts per partner | **N** | 25 | 25 | 25 | 25 | 25 | 25 |
| Number of partners | **M** | 4 | 0.004 | 0.01 | 4 | 0.004 | 0.01 |
| Probability |  | 0.00486 | 0.00001 | 0.00000 | 0.02395 | 0.00017 | 0.00003 |
| Total per 1000 |  | 4.86 | 0.01 | 0.000 | 23.95 | 0.17 | 0.03 |
| Proportion already infected | **I** | 0.20 | 0.20 | 0.20 | 0.08 | 0.08 | 0.08 |
| Total per 1000 - I adjusted |  | 3.89 | 0.01 | 0.000 | 22.03 | 0.16 | 0.02 |
|  |  |  |  | **3.9** |  |  | **22.2** |

**Impact of VCT intervention on risk behaviour** ׀׀

Reduction in condom non-use: 24%

Reduction in partners: 0%

Reduction in STI non-treatment: 0%

**Annual number of new HIV infection averted among 1,000 men with VCT intervention = 5.4**

(Infections occurring in base case scenario minus infections occurring with intervention)

**Women**

**Annual new HIV infections – base scenario without intervention**

|  |  | **Risk of acquiring HIV** | | | **Risk of transmitting HIV** | | |
| --- | --- | --- | --- | --- | --- | --- | --- |
|  |  | **Receptive vaginal** | **Receptive anal** | **Receptive oral** | **Insertive vaginal** | **Insertive anal** | **Insertive oral** |
| HIV prevalence in sex partners* | **P** | 0.08 | 0.08 | 0.08 | 0.20 | 0.20 | 0.20 |
| Risk of HIV per unprotected act† | **R** | 0.0020 | 0.0140 | 0.0006 | 0.0010 | 0.0014 | 0.0000 |
| Fraction of acts with condom‡ | **F** | 0.30 | 0.30 | 0.10 | 0.30 | 0.30 | 0.10 |
| Effectiveness of condom§ | **E** | 0.80 | 0.70 | 0.90 | 0.80 | 0.70 | 0.90 |
| Number of acts per partner¶ | **N** | 25 | 25 | 25 | 25 | 25 | 25 |
| Number of partners¶ | **M** | 4 | 0.004 | 0.01 | 4.000 | 0.004 | 0.01 |
| Probability |  | 0.01165 | 0.00008 | 0.00001 | 0.01468 | 0.00002 | 0.00000 |
| Total per 1000 |  | 11.65 | 0.08 | 0.01 | 14.68 | 0.02 | 0.000 |
| Proportion already infected | **I** | 0.20 | 0.20 | 0.20 | 0.08 | 0.08 | 0.08 |
| Total per 1000 - I adjusted |  | 9.32 | 0.06 | 0.01 | 13.51 | 0.02 | 0.000 |
|  |  |  |  | **9.4** |  |  | **13.5** |

**Annual new HIV infections with VCT intervention**

|  |  | **Risk of acquiring HIV** | | | **Risk of transmitting HIV** | | |
| --- | --- | --- | --- | --- | --- | --- | --- |
|  |  | **Receptive vaginal** | **Receptive anal** | **Receptive oral** | **Insertive vaginal** | **Insertive anal** | **Insertive oral** |
| HIV prevalence in sex partners | **P** | 0.08 | 0.08 | 0.08 | 0.20 | 0.20 | 0.20 |
| Risk of HIV per unprotected act | **R** | 0.0020 | 0.0140 | 0.0006 | 0.0010 | 0.0014 | 0.0000 |
| Fraction of acts with condom ׀׀ | **F** | 0.47 | 0.47 | 0 | 0.47 | 0.47 | 0 |
| Effectiveness of condom | **E** | 0.80 | 0.70 | 0.90 | 0.80 | 0.70 | 0.90 |
| Number of acts per partner | **N** | 25 | 25 | 25 | 25 | 25 | 25 |
| Number of partners | **M** | 4 | 0.004 | 0.01 | 4 | 0.004 | 0.01 |
| Probability |  | 0.00963 | 0.00007 | 0.00001 | 0.01212 | 0.00002 | 0.00000 |
| Total per 1000 |  | 9.63 | 0.07 | 0.01 | 12.12 | 0.02 | 0.00 |
| Proportion already infected | **I** | 0.20 | 0.20 | 0.20 | 0.08 | 0.08 | 0.08 |
| Total per 1000 - I adjusted |  | 7.71 | 0.05 | 0.01 | 11.15 | 0.02 | 0.00 |
|  |  |  |  | **7.8** |  |  | **11.2** |

**Impact of VCT intervention on risk behaviour** ׀׀

Reduction in condom non-use: 24%

Reduction in partners: 0%

Reduction in STI non-treatment: 0%

**Annual number of new HIV infection averted among 1,000 women with VCT intervention = 4.0**

(Infections occurring in base scenario minus infection occurring with intervention)

**Annual number of new HIV infections averted among 1,000 VCT clients (weighted for men 0.70 and women 0.30)** = 5.0**

*HIV prevalence among VCT men clients was estimated as 20% from VCT programme data. HIV prevalence of 8% was assumed for the partners of men VCT clients based on estimated local trends.

†Risk of HIV per unprotected sex act was based on estimates used by UNAIDS (Epidemiological software and tools, 2005; <http://www.unaids.org/en/HIV_data/Epidemiology/episoftware.asp>), or if some risk values were not available from this source they were adapted from published literature. The risk of acquiring HIV in receptive vaginal sex was considered two times higher than insertive vaginal sex, in receptive anal sex five times higher than receptive vaginal sex, in insertive anal sex 1.5 times higher than insertive vaginal sex, in receptive oral sex six times less than receptive vaginal sex, and in insertive oral sex ten times less than receptive oral sex. The risk of transmission was considered three times higher with STI than without STI. We assumed that 20% of all sex acts of VCT clients were with STI in the base scenario, which is based on estimated local trends. The combined transmission probability of unprotected sex acts is therefore weighted for 20% acts with STI and 80% acts without STI in the base scenario.

‡Condom use among the men and women clients attending VCT centres was estimated as 30% of the sex acts in the base case scenario (unpublished programme trends). The fraction of acts with condom for oral sex was assumed half that of vaginal or anal sex.

§Effectiveness of condom against HIV transmission was estimated to be 80% for vaginal sex (Weller and Davis, Cochrane Database Syst Rev 2002;1:CD003255). We assumed effectiveness of condom for anal sex to be 70% and for oral sex to be 90% (Dandona et al, BMC Public Health 2006;6:31).

¶Number of acts per partner and number of partners were adapted from data from a population-based study in Andhra Pradesh (Dandona et al, BMC Medicine 2006;4:31 and Dandona et al, International Journal of Epidemiology 2008;37:1274-86).

׀׀Impact of VCT intervention adapted from literature (Bollinger et al, Studies in Family Planning 2004;35:27-38; Stover et al, Science 2006;311:1474-76).

**Proportion of men and women using VCT centres from programme data.

**Sexually Transmitted Infection (STI) clinics**

**Men**

**Annual new HIV infections – base scenario without intervention**

|  |  | **Risk of acquiring HIV** | | | **Risk of transmitting HIV** | | |
| --- | --- | --- | --- | --- | --- | --- | --- |
|  |  | **Insertive vaginal** | **Insertive anal** | **Insertive oral** | **Receptive vaginal** | **Receptive anal** | **Receptive oral** |
| HIV prevalence in sex partners* | **P** | 0.08 | 0.08 | 0.08 | 0.20 | 0.20 | 0.20 |
| Risk of HIV per unprotected act† | **R** | 0.0021 | 0.0030 | 0.0000 | 0.0042 | 0.0300 | 0.0012 |
| Fraction of acts with condom‡ | **F** | 0.40 | 0.40 | 0.10 | 0.40 | 0.40 | 0.10 |
| Effectiveness of condom§ | **E** | 0.80 | 0.70 | 0.90 | 0.80 | 0.70 | 0.90 |
| Number of acts per partner¶ | **N** | 20 | 20 | 20 | 20 | 20 | 20 |
| Number of partners¶ | **M** | 4 | 0.004 | 0.010 | 4 | 0.004 | 0.010 |
| Probability |  | 0.00899 | 0.00001 | 0.00000 | 0.04374 | 0.00029 | 0.00004 |
| Total per 1000 |  | 8.99 | 0.01 | 0 | 43.74 | 0.29 | 0.04 |
| Proportion already infected | **I** | 0.20 | 0.20 | 0.20 | 0.08 | 0.08 | 0.08 |
| Total per 1000 - I adjusted |  | 7.19 | 0.01 | 0.00 | 40.24 | 0.27 | 0.04 |
|  |  |  |  | **7.2** |  |  | **40.6** |

**Annual new HIV infections with STI clinic intervention**

|  |  | **Risk of acquiring HIV** | | | **Risk of transmitting HIV** | | |
| --- | --- | --- | --- | --- | --- | --- | --- |
|  |  | **Insertive vaginal** | **Insertive anal** | **Insertive oral** | **Receptive vaginal** | **Receptive anal** | **Receptive oral** |
| HIV prevalence in sex partners | **P** | 0.08 | 0.08 | 0.08 | 0.20 | 0.20 | 0.20 |
| Risk of HIV per unprotected act ׀׀ | **R** | 0.0011 | 0.0015 | 0.0000 | 0.0021 | 0.0150 | 0.0006 |
| Fraction of acts with condom ׀׀ | **F** | 0.47 | 0.47 | 0.15 | 0.47 | 0.47 | 0.15 |
| Effectiveness of condom | **E** | 0.80 | 0.70 | 0.90 | 0.80 | 0.70 | 0.90 |
| Number of acts per partner | **N** | 20 | 20 | 20 | 20 | 20 | 20 |
| Number of partners | **M** | 4.000 | 0.004 | 0.010 | 4 | 0.004 | 0.010 |
| Probability |  | 0.00415 | 0.00001 | 0.00000 | 0.02050 | 0.00015 | 0.00002 |
| Total per 1000 |  | 4.15 | 0.01 | 0.000 | 20.50 | 0.15 | 0.02 |
| Proportion already infected | **I** | 0.20 | 0.20 | 0.20 | 0.08 | 0.08 | 0.08 |
| Total per 1000 - I adjusted |  | 3.32 | 0.01 | 0.00 | 18.86 | 0.14 | 0.02 |
|  |  |  |  | **3.3** |  |  | **19.0** |

**Impact of STI intervention on risk behaviour** ׀׀

Reduction in condom non-use: 12%

Reduction in partners: 0%

Reduction in STI non-treatment: 75%

**Annual number of new HIV infection averted among 1,000 men with STI clinic intervention = 25.4**

(Infections occurring in base scenario minus infection occurring with intervention)

**Women**

**Annual new HIV infections – base scenario without intervention**

|  |  | **Risk of acquiring HIV** | | | **Risk of transmitting HIV** | | |
| --- | --- | --- | --- | --- | --- | --- | --- |
|  |  | **Receptive vaginal** | **Receptive anal** | **Receptive oral** | **Insertive vaginal** | **Insertive anal** | **Insertive oral** |
| HIV prevalence in sex partners* | **P** | 0.08 | 0.08 | 0.08 | 0.20 | 0.20 | 0.20 |
| Risk of HIV per unprotected act† | **R** | 0.0042 | 0.0300 | 0.0012 | 0.0021 | 0.0030 | 0.0000 |
| Fraction of acts with condom‡ | **F** | 0.40 | 0.4 | 0.10 | 0.4 | 0.4 | 0.10 |
| Effectiveness of condom§ | **E** | 0.80 | 0.70 | 0.90 | 0.80 | 0.70 | 0.90 |
| Number of acts per partner¶ | **N** | 20 | 20 | 20 | 20 | 20 | 20 |
| Number of partners¶ | **M** | 4 | 0.004 | 0.01 | 4.000 | 0.004 | 0.01 |
| Probability |  | 0.01767 | 0.00011 | 0.00002 | 0.02235 | 0.00003 | 0.00000 |
| Total per 1000 |  | 18 | 0.11 | 0.02 | 22 | 0.03 | 0.000 |
| Proportion already infected | **I** | 0.20 | 0.20 | 0.20 | 0.08 | 0.08 | 0.08 |
| Total per 1000 - I adjusted |  | 14 | 0.09 | 0.01 | 21 | 0.03 | 0.000 |
|  |  |  |  | **14.2** |  |  | **20.6** |

**Annual new HIV infections with STI intervention**

|  |  | **Risk of acquiring HIV** | | | **Risk of transmitting HIV** | | |
| --- | --- | --- | --- | --- | --- | --- | --- |
|  |  | **Receptive vaginal** | **Receptive anal** | **Receptive oral** | **Insertive vaginal** | **Insertive anal** | **Insertive oral** |
| HIV prevalence in sex partners | **P** | 0.08 | 0.08 | 0.08 | 0.20 | 0.20 | 0.20 |
| Risk of HIV per unprotected act ׀׀ | **R** | 0.0021 | 0.0150 | 0.0006 | 0.0011 | 0.0015 | 0.0000 |
| Fraction of acts with condom ׀׀ | **F** | 0.47 | 0.472 | 0 | 0.472 | 0.472 | 0 |
| Effectiveness of condom | **E** | 0.80 | 0.70 | 0.90 | 0.80 | 0.70 | 0.90 |
| Number of acts per partner | **N** | 20 | 20 | 20 | 20 | 20 | 20 |
| Number of partners | **M** | 4 | 0.004 | 0.010 | 4 | 0.004 | 0.010 |
| Probability |  | 0.00824 | 0.00006 | 0.00001 | 0.01035 | 0.00002 | 0.00000 |
| Total per 1000 |  | 8 | 0.06 | 0.010 | 10 | 0.02 | 0.000 |
| Proportion already infected | **I** | 0.20 | 0.20 | 0.20 | 0.08 | 0.08 | 0.08 |
| Total per 1000 - I adjusted |  | 7 | 0.05 | 0.01 | 10 | 0.01 | 0.000 |
|  |  |  |  | **6.6** |  |  | **9.5** |

**Impact of STI intervention on risk behaviour** ׀׀

Reduction in condom non-use: 12%

Reduction in partners: 0%

Reduction in STI non-treatment: 75%

**Annual number of new HIV infection averted among 1,000 women with STI clinic intervention = 18.7**

(Infections occurring in base scenario minus infection occurring with intervention)

**Annual number of new HIV infection averted among 1,000 clients with STI clinic intervention (weighted for men 0.48 and women 0.52)** = 19.8**

[This number of HIV infections averted is 90% of the calculated number as 10% of the infections averted estimated to be attributable to the effect of other interventions on reduction of STI non-treatment through these public sector clinics]

*HIV prevalence among STI clinic men clients was estimated as 20% from programme data. HIV prevalence of 8% was assumed for the partners of men STI clinic clients based on estimated local trends.

†Risk of HIV per unprotected sex act was based on estimates used by UNAIDS (Epidemiological software and tools, 2005; <http://www.unaids.org/en/HIV_data/Epidemiology/episoftware.asp>), or if some risk values were not available from this source they were adapted from published literature. The risk of acquiring HIV in receptive vaginal sex was considered two times higher than insertive vaginal sex, in receptive anal sex five times higher than receptive vaginal sex, in insertive anal sex 1.5 times higher than insertive vaginal sex, in receptive oral sex six times less than receptive vaginal sex, and in insertive oral sex ten times less than receptive oral sex. The risk of transmission was considered three times higher with STI than without STI. We assumed that all sex acts of STI clinic clients were with STI in the base scenario.

‡Condom use among the men and women clients attending STI clinics was estimated as 40% of the sex acts in the base case scenario (unpublished programme trends). The fraction of acts with condom for oral sex was assumed half that of vaginal or anal sex.

§Effectiveness of condom against HIV transmission was estimated to be 80% for vaginal sex (Weller and Davis, Cochrane Database Syst Rev 2002;1:CD003255). We assumed effectiveness of condom for anal sex to be 70% and for oral sex to be 90% (Dandona et al, BMC Public Health 2006;6:31).

¶Number of acts per partner and number of partners were adapted from data from a population-based study in Andhra Pradesh (Dandona et al, BMC Medicine 2006;4:31 and Dandona et al, International Journal of Epidemiology 2008;37:1274-86).

׀׀Impact of intervention on reduction in STI non-treatment was estimated as 75% taking into account a 25% treatment failure or loss to follow-up rate based on local programme trends. We assumed that the impact on condom non-use due to counselling in STI clinic would be half that of VCT intervention.

**Proportion of men and women using STI clinics from programme data.

**Women sex worker (SW) programmes**

**Women sex workers**

**Annual new HIV infections – base scenario without intervention**

|  |  | **Risk of acquiring HIV** | | | **Risk of transmitting HIV** | | |
| --- | --- | --- | --- | --- | --- | --- | --- |
|  |  | **Receptive vaginal** | **Receptive anal** | **Receptive oral** | **Insertive vaginal** | **Insertive anal** | **Insertive oral** |
| HIV prevalence in sex partners* | **P** | 0.04 | 0.04 | 0.04 | 0.12 | 0.12 | 0.12 |
| Risk of HIV per unprotected act† | **R** | 0.0020 | 0.0140 | 0.0006 | 0.0010 | 0.0014 | 0.0000 |
| Fraction of acts with condom‡ | **F** | 0.6 | 0.6 | 0 | 0.6 | 0.6 | 0 |
| Effectiveness of condom§ | **E** | 0.80 | 0.70 | 0.90 | 0.80 | 0.70 | 0.90 |
| Number of acts per partner‡ | **N** | 2.5 | 2.5 | 2.5 | 2.5 | 2.5 | 2.5 |
| Number of partners‡ | **M** | 172 | 0.172 | 0.43 | 1 | 0.001 | 0.0025 |
| Probability |  | 0.01737 | 0.00014 | 0.00002 | 0.00015 | 0.00000 | 0.00000 |
| Total per 1000 |  | 17 | 0.14 | 0.024 | 0 | 0.00 | 0.00 |
| Proportion already infected | **I** | 0.12 | 0.12 | 0.12 | 0.04 | 0.04 | 0.04 |
| Total per 1000 - I adjusted |  | 15.3 | 0.12 | 0.02 | 0.1 | 0.00 | 0.00 |
|  |  |  |  | **15.42** |  |  | **0.15** |

**Annual new HIV infections with SW intervention**

|  |  | **Risk of acquiring HIV** | | | **Risk of transmitting HIV** | | |
| --- | --- | --- | --- | --- | --- | --- | --- |
|  |  | **Receptive vaginal** | **Receptive anal** | **Receptive oral** | **Insertive vaginal** | **Insertive anal** | **Insertive oral** |
| HIV prevalence in sex partners | **P** | 0.04 | 0.04 | 0.04 | 0.12 | 0.12 | 0.12 |
| Risk of HIV per unprotected act¶ | **R** | 0.0017 | 0.0120 | 0.0005 | 0.0008 | 0.0012 | 0.0000 |
| Fraction of acts with condom¶ | **F** | 0.78 | 0.776 | 0 | 0.776 | 0.776 | 0 |
| Effectiveness of condom | **E** | 0.80 | 0.70 | 0.90 | 0.80 | 0.70 | 0.90 |
| Number of acts per partner | **N** | 2.5 | 2.5 | 2.5 | 2.5 | 2.5 | 2.5 |
| Number of partners¶ | **M** | 153 | 0.153 | 0.383 | 1 | 0.001 | 0.003 |
| Probability |  | 0.00970 | 0.00008 | 0.00002 | 0.00010 | 0.00000 | 0.00000 |
| Total per 1000 |  | 10 | 0.08 | 0.02 | 0 | 0.00 | 0.00 |
| Proportion already infected | **I** | 0.12 | 0.12 | 0.12 | 0.04 | 0.04 | 0.04 |
| Total per 1000 - I adjusted |  | 8.5 | 0.07 | 0.02 | 0.1 | 0.00 | 0 |
|  |  |  |  | **8.62** |  |  | **0.09** |

**Impact of SW intervention on risk behaviour**¶

Reduction in condom non-use: 44%

Reduction in partners: 11%

Reduction in STI non-treatment: 50%

**Annual number of new HIV infection averted among sex workers and their clients for 1,000 women sex workers receiving intervention = 18.0**

(Infections occurring in base scenario minus infections occurring with intervention calculated as follows. As each sex worker is estimated to have 172 clients annually without intervention, the number of infections transmitted to clients of 1000 sex workers in the base scenario would be 0.147x172=25.3 in a year. Adding this to the 15.4 infections acquired by 1000 sex workers in a year, the total number of new annual infections in the base scenario would be 40.7. As each sex worker is estimated to have 153 clients annually with intervention, the number of infections transmitted to clients of 1000 sex workers with intervention would be 0.092x153=14.1 in a year. Adding this to the 8.6 infections acquired by 1000 sex workers in a year, the total number of new annual infections with intervention would be 22.7. This is 18 less than the 40.7 new infections without intervention)

*HIV prevalence among women SW was estimated as 12% from programme data and in the clients of women SW as 4% based on estimated local trends.

†Risk of HIV per unprotected sex act was based on estimates used by UNAIDS (Epidemiological software and tools, 2005; <http://www.unaids.org/en/HIV_data/Epidemiology/episoftware.asp>), or if some risk values were not available from this source they were adapted from published literature. The risk of transmission in receptive vaginal sex was considered two times higher than insertive vaginal sex. The risk of transmission in receptive anal sex was considered five times higher than receptive vaginal sex. The risk of transmission in insertive anal sex was considered 1.5 times higher than insertive vaginal sex. The risk of transmission in receptive oral sex was considered six times less than receptive vaginal sex. The risk of transmission in insertive oral sex was considered ten times less than receptive oral sex. The risk of transmission was considered three times higher with STI than without STI. We assumed that 20% of all sex acts of SWs were with STI in the base scenario, which is based on estimated local trends. The combined transmission probability of unprotected sex acts is therefore weighted for 20% acts with STI and 80% acts without STI in the base scenario.

‡Fraction of acts with condom, number of acts per partner and number of partners were adapted from data from a previous large study of women sex workers in Andhra Pradesh (Dandona et al, BMC Public Health 2005, 5:87).

§Effectiveness of condom against HIV transmission was estimated to be 80% for vaginal sex (Weller and Davis, Cochrane Database Syst Rev 2002;1:CD003255). We assumed effectiveness of condom for anal sex to be 70% and for oral sex to be 90% (Dandona et al, BMC Public Health 2006;6:31).

¶Impact of intervention on reduction in condom non-use and the reduction in partners were adapted from literature (Bollinger et al, Studies in Family Planning 2004;35:27-38; Stover et al, Science 2006;311:1474-76). Intervention impact on reduction in STI non-treatment was based on estimated local trends.

**Other women sex partners of clients of women sex workers**

**Annual new HIV infections – base scenario without intervention**

|  |  | **Risk of other women to acquire HIV** | | |
| --- | --- | --- | --- | --- |
|  |  | **Receptive vaginal** | **Receptive anal** | **Receptive oral** |
| HIV prevalence in sex partners* | **P** | 0.04 | 0.04 | 0.04 |
| Risk of HIV per unprotected act† | **R** | 0.0017 | 0.0120 | 0.0005 |
| Fraction of acts with condom‡ | **F** | 0.15 | 0.15 | 0 |
| Effectiveness of condom§ | **E** | 0.80 | 0.70 | 0.90 |
| Number of acts per partner‡ | **N** | 50 | 50 | 50 |
| Number of partners‡ | **M** | 1 | 0.01 | 0.02 |
| Probability |  | 0.0029 | 0.00017 | 0.00002 |
| Total per 1000 |  | 2.85 | 0.17 | 0.02 |
| Proportion already infected | **I** | 0.04 | 0.04 | 0.04 |
| Total per 1000 clients - I adjusted |  | 2.74 | 0.16 | 0.02 |
|  |  |  |  | **2.92** |

**Annual new HIV infections in with SW intervention**

|  |  | **Risk of other women to acquire HIV** | | |
| --- | --- | --- | --- | --- |
|  |  | **Receptive vaginal** | **Receptive anal** | **Receptive oral** |
| HIV prevalence in sex partners | **P** | 0.04 | 0.04 | 0.04 |
| Risk of HIV per unprotected act | **R** | 0.0017 | 0.0120 | 0.0005 |
| Fraction of acts with condom¶ | **F** | 0.176 | 0.176 | 0.000 |
| Effectiveness of condom | **E** | 0.80 | 0.70 | 0.90 |
| Number of acts per partner | **N** | 50 | 50 | 50 |
| Number of partners | **M** | 1 | 0.01 | 0 |
| Probability |  | 0.00279 | 0.00017 | 0.00002 |
| Total per 1000 |  | 2.79 | 0.17 | 0.02 |
| Proportion already infected | **I** | 0.04 | 0.04 | 0.04 |
| Total per 1000 - I adjusted |  | 2.68 | 0.16 | 0.02 |
|  |  |  |  | **2.85** |

**Impact of SW intervention on risk behaviour of clients for sex with other women**¶

Reduction in condom non-use: 3%

Reduction in partners: 0%

Reduction in STI non-treatment: 0%

**Annual number of new HIV infections averted among 1,000 other women sex partners of clients of sex workers with SW intervention = 0.10**

(Infections occurring in base scenario minus infection occurring with intervention)

As each sex worker is estimated to have 153 clients annually with intervention, and assuming that each client of sex workers has on average another woman sex partner, there would be 153 other women benefited for each sex worker with intervention. So, for every 1000 sex workers receiving intervention there would be 14.6 HIV infections averted annually (0.10x153) among other women sex partners of clients of sex workers.

**Total annual number of new HIV infections averted for every 1,000 women sex workers receiving SW intervention = 18.0 in sex workers and their clients plus 14.6 in other woman sex partners of clients = 32.6**

*HIV prevalence among clients of women sex workers was assumed as 4% based on estimated local trends.

†Risk of HIV per unprotected sex act was based on estimates used by UNAIDS (Epidemiological software and tools, 2005; <http://www.unaids.org/en/HIV_data/Epidemiology/episoftware.asp>), or if some risk values were not available from this source they were adapted from published literature. The risk of transmission in receptive vaginal sex was considered two times higher than insertive vaginal sex. The risk of transmission in receptive anal sex was considered five times higher than receptive vaginal sex. The risk of transmission in insertive anal sex was considered 1.5 times higher than insertive vaginal sex. The risk of transmission in receptive oral sex was considered six times less than receptive vaginal sex. The risk of transmission in insertive oral sex was considered ten times less than receptive oral sex. The risk of transmission was considered three times higher with STI than without STI. We assumed that 10% of all sex acts of clients of sex workers with other women were with STI, which is based on estimated local trends. The combined transmission probability of unprotected sex acts is therefore weighted for 10% acts with STI and 90% acts without STI in the base case scenario.

‡Fraction of acts with condom, number of acts per partner and number of partners were adapted from data from a population-based study in Andhra Pradesh (Dandona et al, BMC Medicine 2006;4:31 and Dandona et al, International Journal of Epidemiology 2008;37:1274-86).

§Effectiveness of condom was estimated to be 80% for vaginal sex (Weller and Davis, Cochrane Database Syst Rev 2002;1:CD003255). We assumed effectiveness of condom for anal sex to be 70% and for oral sex to be 90% (Dandona et al, BMC Public Health 2006;6:31).

¶Impact of intervention on reduction in condom non-use by sex worker clients for sex with other women was based on estimated local trends.

**Men who have sex with men (MSM) programmes**

**Sex with men**

**Annual new HIV infections – base scenario without intervention**

|  |  | **Risk of acquiring HIV** | | | **Risk of transmitting HIV** | | |
| --- | --- | --- | --- | --- | --- | --- | --- |
|  |  | **Insertive vaginal** | **Insertive anal** | **Insertive oral** | **Receptive vaginal** | **Receptive anal** | **Receptive oral** |
| HIV prevalence in sex partners* | **P** | 0 | 0.11 | 0.11 | 0 | 0.11 | 0.11 |
| Risk of HIV per unprotected act† | **R** | 0 | 0.0014 | 0 | 0 | 0.0140 | 0.0006 |
| Fraction of acts with condom* | **F** | 0 | 0.50 | 0.50 | 0 | 0.50 | 0.50 |
| Effectiveness of condom‡ | **E** | 0 | 0.70 | 0.90 | 0 | 0.70 | 0.90 |
| Number of acts per partner* | **N** | 0 | 5 | 5 | 0 | 5 | 5 |
| Number of partners* | **M** | 0 | 20 | 20 | 0 | 20 | 20 |
| Probability |  | 0 | 0.0099 | 0.0000 | 0 | 0.0938 | 0.0034 |
| Total per 1000 |  | 0 | 10 | 0.00 | 0 | 94 | 3 |
| Proportion already infected | **I** | 0 | 0.11 | 0.11 | 0 | 0.11 | 0.11 |
| Total per 1000 - I adjusted |  | 0 | 8.9 | 0.00 | 0 | 83.5 | 3.0 |
|  |  |  |  | **8.9** |  |  | **86.5** |

**Annual new HIV infections with MSM intervention**

|  |  | **Risk of acquiring HIV** | | | **Risk of transmitting HIV** | | |
| --- | --- | --- | --- | --- | --- | --- | --- |
|  |  | **Insertive vaginal** | **Insertive anal** | **Insertive oral** | **Receptive vaginal** | **Receptive anal** | **Receptive oral** |
| HIV prevalence in sex partners | **P** | 0 | 0.11 | 0.11 | 0 | 0.11 | 0.11 |
| Risk of HIV per unprotected act§ | **R** | 0 | 0.0012 | 0.0000 | 0 | 0.0124 | 0.0005 |
| Fraction of acts with condom§ | **F** | 0 | 0.70 | 0.70 | 0 | 0.70 | 0.70 |
| Effectiveness of condom | **E** | 0 | 0.70 | 0.90 | 0 | 0.70 | 0.90 |
| Number of acts per partner | **N** | 0 | 5 | 5 | 0 | 5 | 5 |
| Number of partners§ | **M** | 0 | 18 | 18 | 0 | 18 | 18 |
| Probability |  | 0 | 0.0063 | 0.0000 | 0 | 0.0604 | 0.0018 |
| Total per 1000 |  | 0 | 6 | 0.00 | 0 | 60 | 2 |
| Proportion already infected | **I** | 0 | 0.11 | 0.11 | 0 | 0.11 | 0.11 |
| Total per 1000 - I adjusted |  | 0 | 5.6 | 0.00 | 0 | 53.8 | 1.6 |
|  |  |  |  | **5.6** |  |  | **55.4** |

**Impact of MSM intervention on risk behaviour for sex with men**§

Reduction in condom non-use: 39%

Reduction in partners: 10%

Reduction in STI non-treatment: 40%

**Annual number of new HIV infections averted for 1,000 MSM receiving intervention for sex with men = 34.4**

(Infections occurring in base scenario minus infection occurring with intervention)

**MSM sex with women**

**Annual new HIV infections – base scenario without intervention**

|  |  | **Risk of acquiring HIV** | | | **Risk of transmitting HIV** | | |
| --- | --- | --- | --- | --- | --- | --- | --- |
|  |  | **Insertive vaginal** | **Insertive anal** | **Insertive oral** | **Receptive vaginal** | **Receptive anal** | **Receptive oral** |
| HIV prevalence in sex partners* | **P** | 0.06 | 0.06 | 0.06 | 0.11 | 0.11 | 0.11 |
| Risk of HIV per unprotected act† | **R** | 0.0010 | 0.0014 | 0.0000 | 0.0020 | 0.0140 | 0.0006 |
| Fraction of acts with condom* | **F** | 0.25 | 0.25 | 0.25 | 0.25 | 0.25 | 0.25 |
| Effectiveness of condom‡ | **E** | 0.80 | 0.70 | 0.90 | 0.80 | 0.70 | 0.90 |
| Number of acts per partner* | **N** | 33 | 33 | 33 | 33 | 33 | 33 |
| Number of partners* | **M** | 1.5 | 0.002 | 0.004 | 1.5 | 0.002 | 0.004 |
| Probability |  | 0.002 | 0.0000 | 0.0000 | 0.0083 | 0.0001 | 0.0000 |
| Total per 1000 |  | 2 | 0.00 | 0.0000 | 8 | 0.05 | 0.01 |
| Proportion already infected | **I** | 0.11 | 0.11 | 0.11 | 0.06 | 0.06 | 0.06 |
| Total per 1000 - I adjusted |  | 2.0 | 0.00 | 0.000 | 7.8 | 0.05 | 0.01 |
|  |  |  |  | **2.0** |  |  | **7.9** |

**Annual new HIV infections with MSM intervention**

| **MSM with women partners** |  | **Risk of acquiring HIV** | | | **Risk of transmitting HIV** | | |
| --- | --- | --- | --- | --- | --- | --- | --- |
|  |  | **Insertive vaginal** | **Insertive anal** | **Insertive oral** | **Receptive vaginal** | **Receptive anal** | **Receptive oral** |
| HIV prevalence in sex partners | **P** | 0.06 | 0.06 | 0.06 | 0.11 | 0.11 | 0.11 |
| Risk of HIV per unprotected act§ | **R** | 0.0009 | 0.0012 | 0.00000 | 0.0017 | 0.0124 | 0.0005 |
| Fraction of acts with condom§ | **F** | 0.40 | 0.40 | 0.40 | 0.40 | 0.40 | 0.40 |
| Effectiveness of condom | **E** | 0.80 | 0.70 | 0.90 | 0.80 | 0.70 | 0.90 |
| Number of acts per partner | **N** | 33 | 33 | 33 | 33 | 33 | 33 |
| Number of partners§ | **M** | 1.4 | 0.001 | 0.004 | 1.4 | 0.001 | 0.004 |
| Probability |  | 0.002 | 0.000002 | 0.000 | 0.006 | 0.0000 | 0.000004 |
| Total per 1000 |  | 2 | 0.00 | 0.000 | 6 | 0.04 | 0.00 |
| Proportion already infected | **I** | 0.11 | 0.11 | 0.11 | 0.06 | 0.06 | 0.06 |
| Total per 1000 - I adjusted |  | 1.5 | 0.00 | 0.0000 | 5.6 | 0.04 | 0.00 |
|  |  |  |  | **1.5** |  |  | **5.7** |

**Impact of MSM intervention on risk behaviour for sex with women**§

Reduction in condom non-use: 20%

Reduction in partners: 5%

Reduction in STI non-treatment: 40%

**Annual number of new HIV infections averted for 1,000 MSM receiving intervention for sex with women = 2.8**

(Infections occurring in base scenario minus infection occurring with intervention)

**Annual number of new HIV infections averted for 1,000 MSM receiving intervention for sex with men and women = 33.4**

[This number of HIV infections averted is 90% of the calculated number as we estimated that for 40% of the man-to-man sex acts both men would have received the intervention and for these the intervention effect would be a reduced to 1.5 times instead of the two times if each man were counted separately]

*HIV prevalence in MSM and their women sex partners, fraction of acts with condoms, number of acts per partner and number of partners were adapted from data from a population-based study in Andhra Pradesh (Dandona et al, BMC Medicine 2006;4:31 and Dandona et al, International Journal of Epidemiology 2008;37:1274-86) and a large study of MSM in Andhra Pradesh (Dandona et al, AIDS 2005;19:611-19; Dandona et al, BMC Public Health 2006;6:31).

†Risk of HIV per unprotected sex act was based on estimates used by UNAIDS (Epidemiological software and tools, 2005; <http://www.unaids.org/en/HIV_data/Epidemiology/episoftware.asp>), or if some risk values were not available from this source they were adapted from published literature. The risk of transmission in receptive vaginal sex was considered two times higher than insertive vaginal sex. The risk of transmission in receptive anal sex was considered five times higher than receptive vaginal sex. The risk of transmission in insertive anal sex was considered 1.5 times higher than insertive vaginal sex. The risk of transmission in receptive oral sex was considered six times less than receptive vaginal sex. The risk of transmission in insertive oral sex was considered ten times less than receptive oral sex. The risk of transmission was considered three times higher with STI than without STI. We assumed that 20% of all sex acts of MSM were with STI, which is based on estimated local trends. The combined transmission probability of risky unprotected sex acts is therefore weighted for 20% acts with STI and 80% acts without STI in the base scenario.

‡Effectiveness of condom was estimated to be 80% for vaginal sex (Weller and Davis, Cochrane Database Syst Rev 2002;1:CD003255). We assumed effectiveness of condom for anal sex to be 70% and for oral sex to be 90% (Dandona et al, BMC Public Health 2006;6:31).

§Impact of intervention on reduction in condom non-use and reduction in partners for sex with men was adapted from literature (Bollinger et al, Studies in Family Planning 2004;35:27-38; Stover et al, Science 2006;311:1474-76). Intervention impact on reduction in condom non-use and reduction in partners for sex with women, and in STI non-treatment for sex with men and women, was based on estimated local trends.

**Trucker programmes**

**Annual new HIV infections – base scenario without intervention**

| **Truckers** |  | **Risk of acquiring HIV** | | | **Risk of transmitting HIV** | | |
| --- | --- | --- | --- | --- | --- | --- | --- |
|  |  | **Insertive vaginal** | **Insertive anal** | **Insertive oral** | **Receptive vaginal** | **Receptive anal** | **Receptive oral** |
| HIV prevalence in sex partners* | **P** | 0.04 | 0.04 | 0.04 | 0.04 | 0.04 | 0.04 |
| Risk of HIV per unprotected act† | **R** | 0.0008 | 0.0012 | 0.0000 | 0.0016 | 0.0116 | 0.0005 |
| Fraction of acts with condom* | **F** | 0.25 | 0.25 | 0.10 | 0.25 | 0.25 | 0.10 |
| Effectiveness of condom‡ | **E** | 0.80 | 0.70 | 0.90 | 0.80 | 0.70 | 0.90 |
| Number of acts per partner* | **N** | 25 | 25 | 25 | 25 | 25 | 25 |
| Number of partners* | **M** | 4 | 0.004 | 0.01 | 4 | 0.004 | 0.01 |
| Probability |  | 0.00258 | 0.00000 | 0.00000 | 0.00511 | 0.00003 | 0.00000 |
| Total per 1000 |  | 3 | 0.00 | 0.000 | 5 | 0.03 | 0.00 |
| Proportion already infected | **I** | 0.04 | 0.04 | 0.04 | 0.04 | 0.04 | 0.04 |
| Total per 1000 - I adjusted |  | 2.5 | 0.00 | 0.000 | 4.9 | 0.03 | 0.00 |
|  |  |  |  | **2.5** |  |  | **4.9** |

**Annual new HIV infections with trucker intervention**

| **Truckers** |  | **Risk of acquiring HIV** | | | **Risk of transmitting HIV** | | |
| --- | --- | --- | --- | --- | --- | --- | --- |
|  |  | **Insertive vaginal** | **Insertive anal** | **Insertive oral** | **Receptive vaginal** | **Receptive anal** | **Receptive oral** |
| HIV prevalence in sex partners | **P** | 0.04 | 0.04 | 0.04 | 0.04 | 0.04 | 0.04 |
| Risk of HIV per unprotected act§ | **R** | 0.0008 | 0.0011 | 0.0000 | 0.0016 | 0.0113 | 0.0005 |
| Fraction of acts with condom§ | **F** | 0.42 | 0.42 | 0.30 | 0.42 | 0.42 | 0.30 |
| Effectiveness of condom | **E** | 0.80 | 0.70 | 0.90 | 0.80 | 0.70 | 0.90 |
| Number of acts per partner | **N** | 25 | 25 | 25 | 25 | 25 | 25 |
| Number of partners | **M** | 4 | 0.00 | 0.01 | 4 | 0.00 | 0.01 |
| Probability |  | 0.00209 | 0.00000 | 0.00000 | 0.00416 | 0.00003 | 0.00000 |
| Total per 1000 |  | 2 | 0.00 | 0.0000 | 4 | 0.03 | 0.003 |
| Proportion already infected | **I** | 0.04 | 0.04 | 0.04 | 0.04 | 0.04 | 0.04 |
| Total per 1000 - I adjusted |  | 2.0 | 0.00 | 0.000 | 4.0 | 0.03 | 0.003 |
|  |  |  |  | **2.0** |  |  | **4.0** |

**Impact of trucker intervention on risk behaviour**§

Reduction in condom non-use: 22%

Reduction in partners: 0%

Reduction in STI non-treatment: 20%

**Annual number of new HIV infection averted for 1,000 truckers receiving intervention = 1.4**

(Infections occurring in base scenario minus infection occurring with intervention)

*HIV prevalence in truckers and partners of truckers, fraction of acts with condom, number of acts per partner and number of partners (including sex workers, spouse and regular partners) in base scenario were adapted from data from a population-based study in Andhra Pradesh (Dandona et al, BMC Medicine 2006;4:31 and Dandona et al, International Journal of Epidemiology 2008;37:1274-86).

†Risk of HIV per unprotected sex act was based on estimates used by UNAIDS (Epidemiological software and tools, 2005; <http://www.unaids.org/en/HIV_data/Epidemiology/episoftware.asp>), or if some risk values were not available from this source they were adapted from published literature. The risk of transmission in receptive vaginal sex was considered two times higher than insertive vaginal sex. The risk of transmission in receptive anal sex was considered five times higher than receptive vaginal sex. The risk of transmission in insertive anal sex was considered 1.5 times higher than insertive vaginal sex. The risk of transmission in receptive oral sex was considered six times less than receptive vaginal sex. The risk of transmission in insertive oral sex was considered ten times less than receptive oral sex. The risk of transmission was considered three times higher with STI than without STI. We assumed that 8% of all sex acts of truckers were with STI, which is based on estimates from unpublished local data/trends. The combined transmission probability of risky unprotected sex acts is therefore weighted for 8% acts with STI and 92% acts without STI in the base case scenario.

‡Effectiveness of condom was estimated to be 80% for vaginal sex (Weller and Davis, Cochrane Database Syst Rev 2002;1:CD003255). We assumed effectiveness of condom for anal sex to be 70% and for oral sex to be 90% (Dandona et al, BMC Public Health 2006;6:31).

§We assumed that the intervention impact on condom non-use and reduction in STI non-treatment would be roughly half that of women sex worker intervention based on estimated local trends.

**Composite programmes**

**Men**

**Annual new HIV infections – base scenario without intervention**

|  |  | **Risk of acquiring HIV** | | | **Risk of transmitting HIV** | | |
| --- | --- | --- | --- | --- | --- | --- | --- |
|  |  | **Insertive vaginal** | **Insertive anal** | **Insertive oral** | **Receptive vaginal** | **Receptive anal** | **Receptive oral** |
| HIV prevalence in sex partners* | **P** | 0.04 | 0.04 | 0.04 | 0.04 | 0.04 | 0.04 |
| Risk of HIV per unprotected act† | **R** | 0.0008 | 0.0012 | 0.0000 | 0.0016 | 0.0116 | 0.0005 |
| Fraction of acts with condom* | **F** | 0.20 | 0.20 | 0.00 | 0.20 | 0.20 | 0.00 |
| Effectiveness of condom‡ | **E** | 0.80 | 0.70 | 0.90 | 0.80 | 0.70 | 0.90 |
| Number of acts per partner* | **N** | 25 | 25 | 25 | 25 | 25 | 25 |
| Number of partners* | **M** | 4 | 0.00 | 0.01 | 4 | 0.00 | 0.01 |
| Probability |  | 0.00270 | 0.00000 | 0.00000 | 0.00536 | 0.00004 | 0.00000 |
| Total per 1000 |  | 3 | 0.00 | 0.0000 | 5 | 0.04 | 0.00 |
| Proportion already infected | **I** | 0.04 | 0.04 | 0.04 | 0.04 | 0.04 | 0.04 |
| Total per 1000 - I adjusted |  | 2.6 | 0.00 | 0.0000 | 5 | 0.03 | 0.00 |
|  |  |  |  | **2.6** |  |  | **5.2** |

**Annual new HIV infections with composite intervention**

|  |  | **Risk of acquiring HIV** | | | **Risk of transmitting HIV** | | |
| --- | --- | --- | --- | --- | --- | --- | --- |
|  |  | **Insertive vaginal** | **Insertive anal** | **Insertive oral** | **Receptive vaginal** | **Receptive anal** | **Receptive oral** |
| HIV prevalence in sex partners | **P** | 0.04 | 0.04 | 0.04 | 0.04 | 0.04 | 0.04 |
| Risk of HIV per unprotected act§ | **R** | 0.0008 | 0.0011 | 0.0000 | 0.0016 | 0.0113 | 0.0005 |
| Fraction of acts with condom§ | **F** | 0.36 | 0.36 | 0.00 | 0.36 | 0.36 | 0.00 |
| Effectiveness of condom | **E** | 0.80 | 0.70 | 0.90 | 0.80 | 0.70 | 0.90 |
| Number of acts per partner | **N** | 25 | 25 | 25 | 25 | 25 | 25 |
| Number of partners§ | **M** | 4 | 0.00 | 0.01 | 4 | 0.00 | 0.01 |
| Probability |  | 0.00212 | 0.00000 | 0.00000 | 0.00421 | 0.00003 | 0.00000 |
| Total per 1000 |  | 2 | 0.00 | 0.0000 | 4 | 0.03 | 0.00 |
| Proportion already infected | **I** | 0.04 | 0.04 | 0.04 | 0.04 | 0.04 | 0.04 |
| Total per 1000 - I adjusted |  | 2 | 0.003 | 0.0000 | 4.0 | 0.03 | 0.00 |
|  |  |  |  | **2.0** |  |  | **4.1** |

**Impact of composite intervention on risk behaviour**§

Reduction in condom non-use: 20%

Reduction in partners: 5%

Reduction in STI non-treatment: 20%

**Annual number of new HIV infection averted among 1,000 men receiving composite intervention = 1.7**

(Infections occurring in base scenario minus infection occurring with intervention)

**Women**

**Annual new HIV infections – base scenario without intervention**

|  |  | **Risk of acquiring HIV** | | | **Risk of transmitting HIV** | | |
| --- | --- | --- | --- | --- | --- | --- | --- |
|  |  | **Receptive vaginal** | **Receptive anal** | **Receptive oral** | **Insertive vaginal** | **Insertive anal** | **Insertive oral** |
| HIV prevalence in sex partners* | **P** | 0.03 | 0.03 | 0.03 | 0.06 | 0.06 | 0.06 |
| Risk of HIV per unprotected act† | **R** | 0.0016 | 0.0116 | 0.0005 | 0.0008 | 0.0012 | 0.0000 |
| Fraction of acts with condom* | **F** | 0.30 | 0.30 | 0 | 0.30 | 0.30 | 0 |
| Effectiveness of condom‡ | **E** | 0.80 | 0.70 | 0.90 | 0.80 | 0.70 | 0.90 |
| Number of acts per partner* | **N** | 15 | 15 | 15 | 15 | 15 | 15 |
| Number of partners* | **M** | 8 | 0.01 | 0.02 | 8 | 0.01 | 0.02 |
| Probability |  | 0.00440 | 0.00003 | 0.00000 | 0.00442 | 0.00001 | 0.00000 |
| Total per 1000 |  | 4 | 0.03 | 0.004 | 4 | 0.01 | 0.00 |
| Proportion already infected | **I** | 0.06 | 0.06 | 0.06 | 0.03 | 0.03 | 0.03 |
| Total per 1000 - I adjusted |  | 4.1 | 0.03 | 0.004 | 4.3 | 0.01 | 0.000 |
|  |  |  |  | **4.2** |  |  | **4.3** |

**Annual new HIV infections with composite intervention**

|  |  | **Risk of acquiring HIV** | | | **Risk of transmitting HIV** | | |
| --- | --- | --- | --- | --- | --- | --- | --- |
|  |  | **Receptive vaginal** | **Receptive anal** | **Receptive oral** | **Insertive vaginal** | **Insertive anal** | **Insertive oral** |
| HIV prevalence in sex partners | **P** | 0.03 | 0.03 | 0.03 | 0.06 | 0.06 | 0.06 |
| Risk of HIV per unprotected act§ | **R** | 0.0016 | 0.0113 | 0.0005 | 0.0008 | 0.0011 | 0.0000 |
| Fraction of acts with condom§ | **F** | 0.44 | 0.44 | 0 | 0.44 | 0.44 | 0 |
| Effectiveness of condom | **E** | 0.80 | 0.70 | 0.90 | 0.80 | 0.70 | 0.90 |
| Number of acts per partner | **N** | 15 | 15 | 15 | 15 | 15 | 15 |
| Number of partners§ | **M** | 8 | 0.008 | 0.019 | 8 | 0.008 | 0.02 |
| Probability |  | 0.00347 | 0.00003 | 0.00000 | 0.00348 | 0.00001 | 0.00000 |
| Total per 1000 |  | 3 | 0.03 | 0.004 | 3 | 0.01 | 0.00 |
| Proportion already infected | **I** | 0.06 | 0.06 | 0.06 | 0.03 | 0.03 | 0.03 |
| Total per 1000 - I adjusted |  | 3.3 | 0.02 | 0.004 | 3.4 | 0.01 | 0.000 |
|  |  |  |  | **3.3** |  |  | **3.4** |

**Impact of composite intervention on risk behaviour**§

Reduction in condom non-use: 20%

Reduction in partners: 5%

Reduction in STI non-treatment: 20%

**Annual number of new HIV infection averted among 1,000 women receiving composite intervention = 1.8**

(Infections occurring in base scenario minus infection occurring with intervention)

**Annual number of new HIV infections averted among 1,000 composite population (weighted for men 0.73 and women 0.27)**¶ **= 1.7**

*Composite intervention includes a mix of population consisting of slum dwellers, construction workers, transport workers, petty vendors, women sex workers and street children. HIV prevalence in men and women in composite intervention, fraction of acts with condom, number of acts per partner and number of partners in base scenario were adapted from programme data, estimated local trends and a population-based study in Andhra Pradesh (Dandona et al, BMC Medicine 2006;4:31 and Dandona et al, International Journal of Epidemiology 2008;37:1274-86).

†Risk of HIV per unprotected sex act was based on estimates used by UNAIDS (Epidemiological software and tools, 2005; <http://www.unaids.org/en/HIV_data/Epidemiology/episoftware.asp>), or if some risk values were not available from this source they were adapted from published literature. The risk of transmission in receptive vaginal sex was considered two times higher than insertive vaginal sex. The risk of transmission in receptive anal sex was considered five times higher than receptive vaginal sex. The risk of transmission in insertive anal sex was considered 1.5 times higher than insertive vaginal sex. The risk of transmission in receptive oral sex was considered six times less than receptive vaginal sex. The risk of transmission in insertive oral sex was considered ten times less than receptive oral sex. The risk of transmission was considered three times higher with STI than without STI. We assumed that 8% of all sex acts of men and women covered by composite intervention were with STI, which is based on estimated local trends. The combined transmission probability of risky unprotected sex acts is therefore weighted for 8% acts with STI and 92% acts without STI in the base case scenario.

‡Effectiveness of condom was estimated to be 80% for vaginal sex (Weller and Davis, Cochrane Database Syst Rev 2002;1:CD003255). We assumed effectiveness of condom for anal sex to be 70% and for oral sex to be 90% (Dandona et al, BMC Public Health 2006;6:31).

§The intervention impact on condom non-use, reduction in partners and reduction in STI non-treatment were estimated based on the mix of the target groups covered by the composite intervention and the estimated local trends.

¶Proportion of men and women covered by composite intervention from programme data.

**Workplace programmes**

**Men**

**Annual new HIV infections – base scenario without intervention**

|  |  | **Risk of acquiring HIV** | | | **Risk of transmitting HIV** | | |
| --- | --- | --- | --- | --- | --- | --- | --- |
|  |  | **Insertive vaginal** | **Insertive anal** | **Insertive oral** | **Receptive vaginal** | **Receptive anal** | **Receptive oral** |
| HIV prevalence in sex partners* | **P** | 0.03 | 0.03 | 0.03 | 0.03 | 0.03 | 0.03 |
| Risk of HIV per unprotected act† | **R** | 0.0008 | 0.0012 | 0.00000 | 0.0016 | 0.0116 | 0.0005 |
| Fraction of acts with condom* | **F** | 0.20 | 0.20 | 0.00 | 0.20 | 0.20 | 0.00 |
| Effectiveness of condom‡ | **E** | 0.80 | 0.70 | 0.90 | 0.80 | 0.70 | 0.90 |
| Number of acts per partner* | **N** | 65 | 65 | 65 | 65 | 65 | 65 |
| Number of partners* | **M** | 1.5 | 0.002 | 0.005 | 1.5 | 0.002 | 0.005 |
| Probability |  | 0.002 | 0.000004 | 0.0000000 | 0.004 | 0.00003 | 0.000004 |
| Total per 1000 |  | 2 | 0.00 | 0.0000 | 4 | 0.03 | 0.00 |
| Proportion already infected | **I** | 0.03 | 0.03 | 0.03 | 0.03 | 0.03 | 0.03 |
| Total per 1000 - I adjusted |  | 1.9 | 0.00 | 0.0000 | 3.7 | 0.03 | 0.004 |
|  |  |  |  | **1.9** |  |  | **3.7** |

**Annual new HIV infections with workplace intervention**

|  |  | **Risk of acquiring HIV** | | | **Risk of transmitting HIV** | | |
| --- | --- | --- | --- | --- | --- | --- | --- |
|  |  | **Insertive vaginal** | **Insertive anal** | **Insertive oral** | **Receptive vaginal** | **Receptive anal** | **Receptive oral** |
| HIV prevalence in sex partners | **P** | 0.03 | 0.03 | 0.03 | 0.03 | 0.03 | 0.03 |
| Risk of HIV per unprotected act§ | **R** | 0.0008 | 0.0011 | 0.0000 | 0.0016 | 0.0113 | 0.0005 |
| Fraction of acts with condom§ | **F** | 0.36 | 0.36 | 0.00 | 0.36 | 0.36 | 0.00 |
| Effectiveness of condom | **E** | 0.80 | 0.70 | 0.90 | 0.80 | 0.70 | 0.90 |
| Number of acts per partner | **N** | 65 | 65 | 65 | 65 | 65 | 65 |
| Number of partners§ | **M** | 1.4 | 0.002 | 0.005 | 1.4 | 0.002 | 0.005 |
| Probability |  | 0.00153 | 0.00000 | 0.00000 | 0.00301 | 0.00002 | 0.00000 |
| Total per 1000 |  | 1.53 | 0.003 | 0.0000 | 3.01 | 0.02 | 0.004 |
| Proportion already infected | **I** | 0.03 | 0.03 | 0.03 | 0.03 | 0.03 | 0.03 |
| Total per 1000 - I adjusted |  | 1.5 | 0.003 | 0.0000 | 2.9 | 0.02 | 0.004 |
|  |  |  |  | **1.5** |  |  | **3.0** |

**Impact of workplace intervention on risk behaviour**§

Reduction in condom non-use: 20%

Reduction in partners: 5%

Reduction in STI non-treatment: 20%

**Annual number of new HIV infections averted for 1,000 men receiving intervention = 1.2**

(Infections occurring in base scenario minus infection occurring with intervention)

**Women**

**Annual new HIV infections – base scenario without intervention**

|  |  | **Risk of acquiring HIV** | | | **Risk of transmitting HIV** | | |
| --- | --- | --- | --- | --- | --- | --- | --- |
|  |  | **Receptive vaginal** | **Receptive anal** | **Receptive oral** | **Insertive vaginal** | **Insertive anal** | **Insertive oral** |
| HIV prevalence in sex partners* | **P** | 0.02 | 0.02 | 0.02 | 0.03 | 0.03 | 0.03 |
| Risk of HIV per unprotected act† | **R** | 0.0016 | 0.0116 | 0.0005 | 0.0008 | 0.0012 | 0.0000 |
| Fraction of acts with condom* | **F** | 0.20 | 0.20 | 0 | 0.20 | 0.20 | 0 |
| Effectiveness of condom‡ | **E** | 0.80 | 0.70 | 0.90 | 0.80 | 0.70 | 0.90 |
| Number of acts per partner* | **N** | 65 | 65 | 65 | 65 | 65 | 65 |
| Number of partners* | **M** | 1.5 | 0.002 | 0.004 | 1.50 | 0.0015 | 0.004 |
| Probability |  | 0.00255 | 0.00001 | 0.00000 | 0.00195 | 0.00000 | 0.00000 |
| Total per 1000 |  | 3 | 0.01 | 0.002 | 2 | 0.003 | 0.0000 |
| Proportion already infected | **I** | 0.03 | 0.03 | 0.03 | 0.02 | 0.02 | 0.02 |
| Total per 1000 - I adjusted |  | 2.5 | 0.01 | 0.002 | 1.9 | 0.003 | 0.0000 |
|  |  |  |  | **2.5** |  |  | **1.9** |

**Annual new HIV infections with work place intervention**

|  |  | **Risk of acquiring HIV** | | | **Risk of transmitting HIV** | | |
| --- | --- | --- | --- | --- | --- | --- | --- |
|  |  | **Receptive vaginal** | **Receptive anal** | **Receptive oral** | **Insertive vaginal** | **Insertive anal** | **Insertive oral** |
| HIV prevalence in sex partners | **P** | 0.02 | 0.02 | 0.02 | 0.03 | 0.03 | 0.03 |
| Risk of HIV per unprotected act§ | **R** | 0.0016 | 0.0113 | 0.0005 | 0.0008 | 0.0011 | 0.0000 |
| Fraction of acts with condom§ | **F** | 0.36 | 0.36 | 0 | 0.36 | 0.36 | 0 |
| Effectiveness of condom | **E** | 0.80 | 0.70 | 0.90 | 0.80 | 0.70 | 0.90 |
| Number of acts per partner | **N** | 65 | 65 | 65 | 65 | 65 | 65 |
| Number of partners§ | **M** | 1.4 | 0.001 | 0.004 | 1.43 | 0.0014 | 0.004 |
| Probability |  | 0.00201 | 0.00001 | 0.00000 | 0.00153 | 0.00000 | 0.00000 |
| Total per 1000 |  | 2 | 0.01 | 0.002 | 1.53 | 0.002 | 0.0000 |
| Proportion already infected | **I** | 0.03 | 0.03 | 0.03 | 0.02 | 0.02 | 0.02 |
| Total per 1000 - I adjusted |  | 1.9 | 0.01 | 0.002 | 1.50 | 0.002 | 0.0000 |
|  |  |  |  | **2.0** |  |  | **1.5** |

**Impact of workplace intervention on risk behaviour**§

Reduction in condom non-use: 20%

Reduction in partners: 5%

Reduction in STI non-treatment: 20%

**Annual number of new HIV infections averted for 1,000 women receiving intervention = 0.9**

(Infections occurring in base scenario minus infection occurring with intervention)

**Annual number of new HIV infections averted for 1,000 men and women receiving intervention (weighted for men 0.61 and women 0.39)**¶ **= 1.1**

*HIV prevalence among the men and women industrial workers served by this intervention, fraction of acts with condom, number of acts per partner and number of partners in base case scenario adapted from programme data, estimated local trends and data from a population-based study in Andhra Pradesh (Dandona et al, BMC Medicine 2006;4:31 and Dandona et al, International Journal of Epidemiology 2008;37:1274-86).

†Risk of HIV per unprotected sex act was based on estimates used by UNAIDS (Epidemiological software and tools, 2005; <http://www.unaids.org/en/HIV_data/Epidemiology/episoftware.asp>), or if some risk values were not available from this source they were adapted from published literature. The risk of transmission in receptive vaginal sex was considered two times higher than insertive vaginal sex. The risk of transmission in receptive anal sex was considered five times higher than receptive vaginal sex. The risk of transmission in insertive anal sex was considered 1.5 times higher than insertive vaginal sex. The risk of transmission in receptive oral sex was considered six times less than receptive vaginal sex. The risk of transmission in insertive oral sex was considered ten times less than receptive oral sex. The risk of transmission was considered three times higher with STI than without STI. We assumed that 8% of all sex acts of workers were with STI, which is based on estimates from unpublished local data/trends. The combined transmission probability of unprotected sex acts is therefore weighted for 8% acts with STI and 92% acts without STI in the base case scenario.

‡Effectiveness of condom was estimated to be 80% for vaginal sex (Weller and Davis, Cochrane Database Syst Rev 2002;1:CD003255). We assumed effectiveness of condom for anal sex to be 70% and for oral sex to be 90% (Dandona et al, BMC Public Health 2006;6:31).

§Impact of intervention on reduction in condom non-use adapted from literature (Bollinger et al, Studies in Family Planning 2004;35:27-38; Stover et al, Science 2006;311:1474-76). The intervention impact on reduction in partners and in STI non-treatment was assumed based on programme data and estimated local trends.

¶Proportion of men and women covered by workplace intervention from programme data.

**Migrant labourer programmes**

**Men**

**Annual new HIV infections – base scenario without intervention**

|  |  | **Risk of acquiring HIV** | | | **Risk of transmitting HIV** | | |
| --- | --- | --- | --- | --- | --- | --- | --- |
|  |  | **Insertive vaginal** | **Insertive anal** | **Insertive oral** | **Receptive vaginal** | **Receptive anal** | **Receptive oral** |
| HIV prevalence in sex partners* | **P** | 0.04 | 0.04 | 0.04 | 0.04 | 0.04 | 0.04 |
| Risk of HIV per unprotected act† | **R** | 0.0008 | 0.0012 | 0.0000 | 0.0016 | 0.0116 | 0.0005 |
| Fraction of acts with condom* | **F** | 0.20 | 0.20 | 0.00 | 0.20 | 0.20 | 0.00 |
| Effectiveness of condom‡ | **E** | 0.80 | 0.70 | 0.90 | 0.80 | 0.70 | 0.90 |
| Number of acts per partner* | **N** | 40 | 40 | 40 | 40 | 40 | 40 |
| Number of partners* | **M** | 2.5 | 0.003 | 0.006 | 2.5 | 0.003 | 0.006 |
| Probability |  | 0.00269 | 0.00000 | 0.00000 | 0.00531 | 0.00003 | 0.00000 |
| Total per 1000 |  | 3 | 0.00 | 0.0000 | 5 | 0.03 | 0.005 |
| Proportion already infected | **I** | 0.04 | 0.04 | 0.04 | 0.04 | 0.04 | 0.04 |
| Total per 1000 - I adjusted |  | 2.6 | 0.00 | 0.000 | 5.1 | 0.03 | 0.004 |
|  |  |  |  | **2.6** |  |  | **5.1** |

**Annual new HIV infections with migrant labourer intervention**

| **Migrant labourers Men** |  | **Risk of acquiring HIV** | | | **Risk of transmitting HIV** | | |
| --- | --- | --- | --- | --- | --- | --- | --- |
|  |  | **Insertive vaginal** | **Insertive anal** | **Insertive oral** | **Receptive vaginal** | **Receptive anal** | **Receptive oral** |
| HIV prevalence in sex partners | **P** | 0.04 | 0.04 | 0.04 | 0.04 | 0.04 | 0.04 |
| Risk of HIV per unprotected act§ | **R** | 0.0008 | 0.0011 | 0.0000 | 0.0016 | 0.0113 | 0.0005 |
| Fraction of acts with condom§ | **F** | 0.36 | 0.36 | 0.00 | 0.36 | 0.36 | 0.00 |
| Effectiveness of condom | **E** | 0.80 | 0.70 | 0.90 | 0.80 | 0.70 | 0.90 |
| Number of acts per partner | **N** | 40 | 40 | 40 | 40 | 40 | 40 |
| Number of partners§ | **M** | 2.4 | 0.002 | 0.006 | 2.4 | 0.002 | 0.006 |
| Probability |  | 0.00211 | 0.00000 | 0.00000 | 0.00418 | 0.00003 | 0.00000 |
| Total per 1000 |  | 2 | 0.00 | 0.0000 | 4 | 0.03 | 0.004 |
| Proportion already infected | **I** | 0.04 | 0.04 | 0.04 | 0.04 | 0.04 | 0.04 |
| Total per 1000 - I adjusted |  | 2.0 | 0.00 | 0.000 | 4.0 | 0.03 | 0.004 |
|  |  |  |  | **2.0** |  |  | **4.0** |

**Impact of migrant labourer intervention on risk behaviour**§

Reduction in condom non-use: 20%

Reduction in partners: 5%

Reduction in STI non-treatment: 20%

**Annual number of new HIV infections averted for 1,000 men migrant labourer receiving intervention = 1.6**

(Infections occurring in base scenario minus infection occurring with intervention)

**Women**

**Annual new HIV infections – base scenario without intervention**

|  |  | **Risk of acquiring HIV** | | | **Risk of transmitting HIV** | | |
| --- | --- | --- | --- | --- | --- | --- | --- |
|  |  | **Receptive vaginal** | **Receptive anal** | **Receptive oral** | **Insertive vaginal** | **Insertive anal** | **Insertive oral** |
| HIV prevalence in sex partners* | **P** | 0.04 | 0.04 | 0.04 | 0.04 | 0.04 | 0.04 |
| Risk of HIV per unprotected act† | **R** | 0.0016 | 0.0116 | 0.0005 | 0.0008 | 0.0012 | 0.0000 |
| Fraction of acts with condom* | **F** | 0.20 | 0.20 | 0.00 | 0.20 | 0.20 | 0.00 |
| Effectiveness of condom‡ | **E** | 0.80 | 0.70 | 0.90 | 0.80 | 0.70 | 0.90 |
| Number of acts per partner* | **N** | 40 | 40 | 40 | 40 | 40 | 40 |
| Number of partners* | **M** | 3 | 0.003 | 0.008 | 3.000 | 0.003 | 0.008 |
| Probability |  | 0.00636 | 0.00004 | 0.00001 | 0.00323 | 0.00000 | 0.00000 |
| Total per 1000 |  | 6 | 0.04 | 0.006 | 3 | 0.005 | 0.0000 |
| Proportion already infected | **I** | 0.04 | 0.04 | 0.04 | 0.04 | 0.04 | 0.04 |
| Total per 1000 - I adjusted |  | 6 | 0.04 | 0.01 | 3.1 | 0.005 | 0.0000 |
|  |  |  |  | **6.2** |  |  | **3.1** |

**Annual new HIV infections with migrant labour intervention**

|  |  | **Risk of acquiring HIV** | | | **Risk of transmitting HIV** | | |
| --- | --- | --- | --- | --- | --- | --- | --- |
|  |  | **Receptive vaginal** | **Receptive anal** | **Receptive oral** | **Insertive vaginal** | **Insertive anal** | **Insertive oral** |
| HIV prevalence in sex partners | **P** | 0.04 | 0.04 | 0.04 | 0.04 | 0.04 | 0.04 |
| Risk of HIV per unprotected act§ | **R** | 0.0016 | 0.0113 | 0.0005 | 0.0008 | 0.0011 | 0.0000 |
| Fraction of acts with condom§ | **F** | 0.36 | 0.36 | 0 | 0.36 | 0.36 | 0 |
| Effectiveness of condom | **E** | 0.80 | 0.70 | 0.90 | 0.80 | 0.70 | 0.90 |
| Number of acts per partner | **N** | 40 | 40 | 40 | 40 | 40 | 40 |
| Number of partners§ | **M** | 2.85 | 0.003 | 0.007 | 2.85 | 0.0029 | 0.007 |
| Probability |  | 0.00501 | 0.00003 | 0.00001 | 0.00253 | 0.00000 | 0.00000 |
| Total per 1000 |  | 5 | 0.03 | 0.005 | 3 | 0.004 | 0.0000 |
| Proportion already infected | **I** | 0.04 | 0.04 | 0.04 | 0.04 | 0.04 | 0.04 |
| Total per 1000 - I adjusted |  | 5 | 0.03 | 0.00 | 2 | 0.004 | 0.0000 |
|  |  |  |  | **4.8** |  |  | **2.4** |

**Impact of migrant labourer intervention on risk behaviour** §

Reduction in condom non-use: 20%

Reduction in partners: 5%

Reduction in STI non-treatment: 20%

**Annual number of new HIV infection averted for 1,000 women migrant labourers receiving intervention = 2**

(Infections occurring in base scenario minus infection occurring with intervention)

**Annual number of new HIV infection averted for 1,000 men and women migrant labourers receiving intervention (weighted for men 0.60 and women 0.40)**¶ **= 1.8**

*HIV prevalence among the men and women migrant labourers, fraction of acts with condom, number of acts per partner and number of partners in base scenario adapted from programme data, estimated local trends and data from a population-based study in Andhra Pradesh (Dandona et al, BMC Medicine 2006;4:31 and Dandona et al, International Journal of Epidemiology 2008;37:1274-86).

†Risk of HIV per unprotected sex act was based on estimates used by UNAIDS (Epidemiological software and tools, 2005; <http://www.unaids.org/en/HIV_data/Epidemiology/episoftware.asp>), or if some risk values were not available from this source they were adapted from published literature. The risk of transmission in receptive vaginal sex was considered two times higher than insertive vaginal sex. The risk of transmission in receptive anal sex was considered five times higher than receptive vaginal sex. The risk of transmission in insertive anal sex was considered 1.5 times higher than insertive vaginal sex. The risk of transmission in receptive oral sex was considered six times less than receptive vaginal sex. The risk of transmission in insertive oral sex was considered ten times less than receptive oral sex. The risk of transmission was considered three times higher with STI than without STI. We assumed that 8% of all sex acts of migrant labourers were with STI, which is based on estimates from unpublished local data/trends. The combined transmission probability of risky unprotected sex acts is therefore weighted for 8% acts with STI and 92% acts without STI in the base case scenario.

‡Effectiveness of condom was estimated to be 80% for vaginal sex (Weller and Davis, Cochrane Database Syst Rev 2002;1:CD003255). We assumed effectiveness of condom for anal sex to be 70% and for oral sex to be 90% (Dandona et al, BMC Public Health 2006;6:31).

§Impact of intervention on reduction in condom non-use adapted from literature (Bollinger et al, Studies in Family Planning 2004;35:27-38; Stover et al, Science 2006;311:1474-76). The intervention impact on reduction in partners and in STI non-treatment was assumed based on programme data and estimated local trends.

¶Proportion of men and women covered by workplace intervention from programme data.

**Street children programmes**

**Boys**

**Annual new HIV – base scenario without intervention**

|  |  | **Risk of acquiring HIV** | | | **Risk of transmitting HIV** | | |
| --- | --- | --- | --- | --- | --- | --- | --- |
|  |  | **Insertive vaginal** | **Insertive anal** | **Insertive oral** | **Receptive vaginal** | **Receptive anal** | **Receptive oral** |
| HIV prevalence in sex partners* | **P** | 0.04 | 0.04 | 0.04 | 0.02 | 0.02 | 0.02 |
| Risk of HIV per unprotected act† | **R** | 0.0008 | 0.0012 | 0.00000 | 0.0016 | 0.0116 | 0.0005 |
| Fraction of acts with condom* | **F** | 0.20 | 0.2 | 0 | 0.2 | 0.2 | 0 |
| Effectiveness of condom‡ | **E** | 0.80 | 0.70 | 0.90 | 0.80 | 0.70 | 0.90 |
| Number of acts per partner* | **N** | 6 | 6 | 6 | 6 | 6 | 6 |
| Number of partners* | **M** | 5 | 6 | 6 | 5 | 6 | 6 |
| Probability |  | 0.0005 | 0.001 | 0.00000 | 0.0005 | 0.005 | 0.00022 |
| Total per 1000 |  | 0.54 | 1 | 0.00 | 0.54 | 5 | 0.22 |
| Proportion already infected | **I** | 0.02 | 0.02 | 0.02 | 0.04 | 0.04 | 0.04 |
| Total per 1000 - I adjusted |  | 0.53 | 0.9 | 0.00 | 0.52 | 4.5 | 0.21 |
|  |  |  |  | **1.5** |  |  | **5.3** |

**Annual new HIV infections with street children intervention**

|  |  | **Risk of acquiring HIV** | | | **Risk of transmitting HIV** | | |
| --- | --- | --- | --- | --- | --- | --- | --- |
|  |  | **Insertive vaginal** | **Insertive anal** | **Insertive oral** | **Receptive vaginal** | **Receptive anal** | **Receptive oral** |
| HIV prevalence in sex partners | **P** | 0.04 | 0.04 | 0.04 | 0.02 | 0.02 | 0.02 |
| Risk of HIV per unprotected act§ | **R** | 0.0008 | 0.0011 | 0.0000 | 0.0016 | 0.0114 | 0.0005 |
| Fraction of acts with condom§ | **F** | 0.28 | 0.28 | 0.00 | 0.28 | 0.28 | 0.00 |
| Effectiveness of condom | **E** | 0.80 | 0.70 | 0.90 | 0.80 | 0.70 | 0.90 |
| Number of acts per partner | **N** | 6 | 6 | 6 | 6 | 6 | 6 |
| Number of partners§ | **M** | 4.8 | 5.7 | 5.7 | 4.8 | 5.7 | 5.7 |
| Probability |  | 0.00047 | 0.00084 | 0.00000 | 0.00047 | 0.00413 | 0.00021 |
| Total per 1000 |  | 0.47 | 0.84 | 0.00 | 0.47 | 4.13 | 0.209 |
| Proportion already infected | **I** | 0.02 | 0.02 | 0.02 | 0.04 | 0.04 | 0.04 |
| Total per 1000 - I adjusted |  | 0.46 | 0.82 | 0.00 | 0.45 | 3.96 | 0.20 |
|  |  |  |  | **1.3** |  |  | **4.6** |

**Impact of street children intervention on risk behaviour**§

Reduction in condom non-use: 10%

Reduction in partners: 5%

Reduction in STI non-treatment: 10%

**Annual number of new HIV infections averted for 1,000 boys receiving intervention = 0.8**

(Infections occurring in base scenario minus infection occurring with intervention)

**Girls**

**Annual new HIV – base scenario without intervention**

|  |  | **Risk of acquiring HIV** | | | **Risk of transmitting HIV** | | |
| --- | --- | --- | --- | --- | --- | --- | --- |
|  |  | **Receptive vaginal** | **Receptive anal** | **Receptive oral** | **Insertive vaginal** | **Insertive anal** | **Insertive oral** |
| HIV prevalence in sex partners* | **P** | 0.04 | 0.04 | 0.04 | 0.04 | 0.04 | 0.04 |
| Risk of HIV per unprotected act† | **R** | 0.0016 | 0.0116 | 0.0005 | 0.0008 | 0.0012 | 0.0000 |
| Fraction of acts with condom* | **F** | 0.2 | 0.2 | 0 | 0.20 | 0.2 | 0 |
| Effectiveness of condom‡ | **E** | 0.80 | 0.70 | 0.90 | 0.80 | 0.70 | 0.90 |
| Number of acts per partner* | **N** | 5 | 5 | 5 | 5 | 5 | 5 |
| Number of partners* | **M** | 10 | 0.01 | 0.025 | 10 | 0.010 | 0.025 |
| Probability |  | 0.00272 | 0.00002 | 0.00000 | 0.00136 | 0.00000 | 0.00000 |
| Total per 1000 |  | 3 | 0.02 | 0.00 | 1 | 0.00 | 0.0000 |
| Proportion already infected | **I** | 0.04 | 0.04 | 0.04 | 0.04 | 0.04 | 0.04 |
| Total per 1000 - I adjusted |  | 2.6 | 0.02 | 0.002 | 1.3 | 0.002 | 0.0000 |
|  |  |  |  | **2.6** |  |  | **1.3** |

**Annual new HIV infections with street children intervention**

|  |  | **Risk of acquiring HIV** | | | **Risk of transmitting HIV** | | |
| --- | --- | --- | --- | --- | --- | --- | --- |
|  |  | **Receptive vaginal** | **Receptive anal** | **Receptive oral** | **Insertive vaginal** | **Insertive anal** | **Insertive oral** |
| HIV prevalence in sex partners | **P** | 0.04 | 0.04 | 0.04 | 0.04 | 0.04 | 0.04 |
| Risk of HIV per unprotected act§ | **R** | 0.0016 | 0.0114 | 0.0005 | 0.0008 | 0.0011 | 0.0000 |
| Fraction of acts with condom§ | **F** | 0.28 | 0.28 | 0.00 | 0.28 | 0.28 | 0.00 |
| Effectiveness of condom | **E** | 0.80 | 0.70 | 0.90 | 0.80 | 0.70 | 0.90 |
| Number of acts per partner | **N** | 5 | 5 | 5 | 5 | 5 | 5 |
| Number of partners§ | **M** | 10 | 0.010 | 0.024 | 10 | 0.010 | 0.024 |
| Probability |  | 0.00235 | 0.00002 | 0.00000 | 0.00118 | 0.00000 | 0.00000 |
| Total per 1000 |  | 2 | 0.02 | 0.002 | 1 | 0.00 | 0.0000 |
| Proportion already infected | **I** | 0.04 | 0.04 | 0.04 | 0.04 | 0.04 | 0.04 |
| Total per 1000 - I adjusted |  | 2 | 0.02 | 0.00 | 1 | 0.00 | 0.0000 |
|  |  |  |  | **2.3** |  |  | **1.1** |

**Impact of street children intervention on risk behaviour**§

Reduction in condom non-use: 10%

Reduction in partners: 5%

Reduction in STI non-treatment: 10%

**Annual number of new HIV infection averted for 1,000 girls receiving intervention = 0.5**

(Infections occurring in base scenario minus infection occurring with intervention)

**Annual number of new HIV infection averted for 1,000 boys and girls receiving intervention (weighted for boys 0.97 and girls 0.03)**¶ **= 0.8**

*HIV prevalence of among the boys and girls, fraction of acts with condom, number of acts per partner and number of partners in base scenario were assumed based on programme data and estimated local trends.

†Risk of HIV per unprotected sex act was based on estimates used by UNAIDS (Epidemiological software and tools, 2005; <http://www.unaids.org/en/HIV_data/Epidemiology/episoftware.asp>), or if some risk values were not available from this source they were adapted from published literature. The risk of transmission in receptive vaginal sex was considered two times higher than insertive vaginal sex. The risk of transmission in receptive anal sex was considered five times higher than receptive vaginal sex. The risk of transmission in insertive anal sex was considered 1.5 times higher than insertive vaginal sex. The risk of transmission in receptive oral sex was considered six times less than receptive vaginal sex. The risk of transmission in insertive oral sex was considered ten times less than receptive oral sex. The risk of transmission was considered three times higher with STI than without STI. We assumed that 8% of all sex acts of street children were with STI, which is based on estimates from unpublished local data/trends. The combined transmission probability of risky unprotected sex acts is therefore weighted for 8% acts with STI and 92% acts without STI in the base case scenario.

‡Effectiveness of condom was estimated to be 80% for vaginal sex (Weller and Davis, Cochrane Database Syst Rev 2002;1:CD003255). We assumed effectiveness of condom for anal sex to be 70% and for oral sex to be 90% (Dandona et al, BMC Public Health 2006;6:31).

§Impact of intervention on reduction in condom non-use, number of partners and in STI non-treatment was assumed based on programme data and estimated local trends.

¶Proportion of boys and girls covered by street children intervention from programme data.

**Prisoners programmes**

**Men**

**Annual new HIV infections in men prisoners – base scenario without intervention**

|  |  | **Risk of acquiring HIV** | | | **Risk of transmitting HIV** | | |
| --- | --- | --- | --- | --- | --- | --- | --- |
|  |  | **Insertive vaginal** | **Insertive anal** | **Insertive oral** | **Receptive vaginal** | **Receptive anal** | **Receptive oral** |
| HIV prevalence in sex partners* | **P** | 0.05 | 0.05 | 0.05 | 0.05 | 0.05 | 0.05 |
| Risk of HIV per unprotected act† | **R** | 0.0008 | 0.0012 | 0.0000 | 0.0016 | 0.0116 | 0.0005 |
| Fraction of acts with condom* | **F** | 0.20 | 0.20 | 0.00 | 0.20 | 0.20 | 0.00 |
| Effectiveness of condom‡ | **E** | 0.80 | 0.70 | 0.90 | 0.80 | 0.70 | 0.90 |
| Number of acts per partner* | **N** | 12 | 12 | 12 | 12 | 12 | 12 |
| Number of partners* | **M** | 6 | 0.01 | 0.02 | 6 | 0.01 | 0.02 |
| Probability |  | 0.00244 | 0.00000 | 0.00000 | 0.00486 | 0.00003 | 0.00000 |
| Total per 1000 |  | 2 | 0.00 | 0.000 | 5 | 0.03 | 0.00 |
| Proportion already infected | **I** | 0.05 | 0.05 | 0.05 | 0.05 | 0.05 | 0.05 |
| Total per 1000 - I adjusted |  | 2.3 | 0.00 | 0.000 | 4.6 | 0.03 | 0.00 |
|  |  |  |  | **2.3** |  |  | **4.7** |

**Annual new HIV infections with prisoners intervention**

|  |  | **Risk of acquiring HIV** | | | **Risk of transmitting HIV** | | |
| --- | --- | --- | --- | --- | --- | --- | --- |
|  |  | **Insertive vaginal** | **Insertive anal** | **Insertive oral** | **Receptive vaginal** | **Receptive anal** | **Receptive oral** |
| HIV prevalence in sex partners | **P** | 0.05 | 0.05 | 0.05 | 0.05 | 0.05 | 0.05 |
| Risk of HIV per unprotected act§ | **R** | 0.0008 | 0.0012 | 0.0000 | 0.0016 | 0.0115 | 0.0005 |
| Fraction of acts with condom§ | **F** | 0.24 | 0.24 | 0.00 | 0.24 | 0.24 | 0.00 |
| Effectiveness of condom | **E** | 0.80 | 0.70 | 0.90 | 0.80 | 0.70 | 0.90 |
| Number of acts per partner | **N** | 12 | 12 | 12 | 12 | 12 | 12 |
| Number of partners | **M** | 6 | 0.01 | 0.02 | 6 | 0.01 | 0.02 |
| Probability |  | 0.00233 | 0.00000 | 0.00000 | 0.00465 | 0.00003 | 0.00000 |
| Total per 1000 |  | 2 | 0.00 | 0.0000 | 5 | 0.03 | 0.00 |
| Proportion already infected | **I** | 0.05 | 0.05 | 0.05 | 0.05 | 0.05 | 0.05 |
| Total per 1000 - I adjusted |  | 2.2 | 0.00 | 0.000 | 4.4 | 0.03 | 0.00 |
|  |  |  |  | **2.2** |  |  | **4.5** |

**Impact of prisoners intervention on risk behaviour**§

Reduction in condom non-use: 5%

Reduction in partners: 0%

Reduction in STI non-treatment: 5%

**Annual number of new HIV infection for 1,000 men prisoners receiving intervention = 0.3**

(Infections occurring in base scenario minus infection occurring with intervention)

**Women**

**Annual new HIV infections – base scenario without intervention**

|  |  | **Risk of acquiring HIV** | | | **Risk of transmitting HIV** | | |
| --- | --- | --- | --- | --- | --- | --- | --- |
|  |  | **Receptive vaginal** | **Receptive anal** | **Receptive oral** | **Insertive vaginal** | **Insertive anal** | **Insertive oral** |
| HIV prevalence in sex partners* | **P** | 0.05 | 0.05 | 0.05 | 0.05 | 0.05 | 0.05 |
| Risk of HIV per unprotected act† | **R** | 0.0016 | 0.0116 | 0.0005 | 0.0008 | 0.0012 | 0.0000 |
| Fraction of acts with condom* | **F** | 0.20 | 0.20 | 0 | 0.200 | 0.20 | 0 |
| Effectiveness of condom‡ | **E** | 0.80 | 0.70 | 0.90 | 0.80 | 0.70 | 0.90 |
| Number of acts per partner* | **N** | 10 | 10 | 10 | 10 | 10 | 10 |
| Number of partners* | **M** | 10 | 0.01 | 0.03 | 10 | 0.01 | 0.03 |
| Probability |  | 0.00676 | 0.00005 | 0.00001 | 0.00339 | 0.00000 | 0.00000 |
| Total per 1000 |  | 7 | 0.05 | 0.006 | 3 | 0.005 | 0.0000 |
| Proportion already infected | **I** | 0.05 | 0.05 | 0.05 | 0.05 | 0.05 | 0.05 |
| Total per 1000 - I adjusted |  | 6.4 | 0.05 | 0.005 | 3.2 | 0.005 | 0.0000 |
|  |  |  |  | **6.5** |  |  | **3.2** |

**Annual new HIV infections with prisoners intervention**

|  |  | **Risk of acquiring HIV** | | | **Risk of transmitting HIV** | | |
| --- | --- | --- | --- | --- | --- | --- | --- |
|  |  | **Receptive vaginal** | **Receptive anal** | **Receptive oral** | **Insertive vaginal** | **Insertive anal** | **Insertive oral** |
| HIV prevalence in sex partners | **P** | 0.05 | 0.05 | 0.05 | 0.05 | 0.05 | 0.05 |
| Risk of HIV per unprotected act§ | **R** | 0.0016 | 0.0115 | 0.0005 | 0.0008 | 0.0012 | 0.00000 |
| Fraction of acts with condom§ | **F** | 0.24 | 0.24 | 0 | 0.24 | 0.24 | 0 |
| Effectiveness of condom | **E** | 0.80 | 0.70 | 0.90 | 0.80 | 0.70 | 0.90 |
| Number of acts per partner | **N** | 10.0 | 10.0 | 10.0 | 10.0 | 10.0 | 10.0 |
| Number of partners | **M** | 10 | 0.010 | 0.025 | 10 | 0.010 | 0.025 |
| Probability |  | 0.006 | 0.00005 | 0.000006 | 0.003 | 0.000005 | 0.0000000 |
| Total per 1000 |  | 6.46 | 0.05 | 0.006 | 3.24 | 0.005 | 0.0000 |
| Proportion already infected | **I** | 0.05 | 0.05 | 0.05 | 0.05 | 0.05 | 0.05 |
| Total per 1000 - I adjusted |  | 6.14 | 0.04 | 0.01 | 3.08 | 0.005 | 0.0000 |
|  |  |  |  | **6.2** |  |  | **3.1** |

**Impact of prisoners intervention on risk behaviour**§

Reduction in condom non-use: 5%

Reduction in partners: 0%

Reduction in STI non-treatment: 5%

**Annual number of new HIV infection averted for 1,000 women prisoners receiving intervention = 0.4**

(Infections occurring in base scenario minus infection occurring with intervention)

**Annual number of new HIV infection averted for 1,000 men and women prisoners receiving intervention (weighted for men 0.95 and women 0.05)**¶ **= 0.3**

*HIV prevalence among the men and women prisoners, fraction of acts with condom, number of acts per partner and number of partners in base scenario adapted from programme data and estimated local trends.

†Risk of HIV per unprotected sex act was based on estimates used by UNAIDS (Epidemiological software and tools, 2005; <http://www.unaids.org/en/HIV_data/Epidemiology/episoftware.asp>), or if some risk values were not available from this source they were adapted from published literature. The risk of transmission in receptive vaginal sex was considered two times higher than insertive vaginal sex. The risk of transmission in receptive anal sex was considered five times higher than receptive vaginal sex. The risk of transmission in insertive anal sex was considered 1.5 times higher than insertive vaginal sex. The risk of transmission in receptive oral sex was considered six times less than receptive vaginal sex. The risk of transmission in insertive oral sex was considered ten times less than receptive oral sex. The risk of transmission was considered three times higher with STI than without STI. We assumed that 8% of all sex acts of prisoners were with STI, which is based on estimates from unpublished local data/trends. The combined transmission probability of risky unprotected sex acts is therefore weighted for 8% acts with STI and 92% acts without STI in the base case scenario.

‡Effectiveness of condom was estimated to be 80% for vaginal sex (Weller and Davis, Cochrane Database Syst Rev 2002;1:CD003255). We assumed effectiveness of condom for anal sex to be 70% and for oral sex to be 90% (Dandona et al, BMC Public Health 2006;6:31).

§Impact of intervention on reduction in reduction in condom non-use and the reduction in STI non-treatment we assumed roughly half the impacts as that of street children intervention.

¶Proportion of men and women covered by prisoners intervention from programme data.

**Information Education and Communication (IEC) for general public**

**Men**

**Annual new HIV infections – base scenario without intervention**

|  | | **Risk of acquiring HIV** | | | **Risk of transmitting HIV** | | |
| --- | --- | --- | --- | --- | --- | --- | --- |
|  |  | **Insertive vaginal** | **Insertive anal** | **Insertive oral** | **Receptive vaginal** | **Receptive anal** | **Receptive oral** |
| HIV prevalence in sex partners* | **P** | 0.010 | 0.010 | 0.010 | 0.012 | 0.012 | 0.012 |
| Risk of HIV per unprotected act† | **R** | 0.0005 | 0.0007 | 0.0000 | 0.0010 | 0.0073 | 0.0003 |
| Fraction of acts with condom‡ | **F** | 0.08 | 0.08 | 0.00 | 0.08 | 0.08 | 0.00 |
| Effectiveness of condom§ | **E** | 0.80 | 0.70 | 0.90 | 0.80 | 0.70 | 0.90 |
| Number of acts per partner‡ | **N** | 70 | 70 | 70 | 70 | 70 | 70 |
| Number of partners‡ | **M** | 1 | 0.001 | 0.003 | 1 | 0.001 | 0.003 |
| Probability |  | 0.00033 | 0.00000 | 0.00000 | 0.00078 | 0.00000 | 0.00000 |
| Total per 1000 |  | 0.33 | 0.000 | 0.0000 | 0.78 | 0.00 | 0.00 |
| Proportion already infected | **I** | 0.01 | 0.01 | 0.01 | 0.01 | 0.01 | 0.01 |
| Total per 1000 - I adjusted |  | 0.32 | 0.000 | 0.00000 | 0.77 | 0.00 | 0.001 |
|  |  |  |  | **0.325** |  |  | **0.773** |

**Annual new HIV infections with IEC intervention**

|  | | **Risk of acquiring HIV** | | | **Risk of transmitting HIV** | | |
| --- | --- | --- | --- | --- | --- | --- | --- |
|  |  | **Insertive vaginal** | **Insertive anal** | **Insertive oral** | **Receptive vaginal** | **Receptive anal** | **Receptive oral** |
| HIV prevalence in sex partners | **P** | 0.010 | 0.010 | 0.010 | 0.012 | 0.012 | 0.012 |
| Risk of HIV per unprotected act | **R** | 0.0005 | 0.0007 | 0.0000 | 0.0010 | 0.0073 | 0.0003 |
| Fraction of acts with condom¶ | **F** | 0.10 | 0.10 | 0.00 | 0.10 | 0.10 | 0.00 |
| Effectiveness of condom | **E** | 0.80 | 0.70 | 0.90 | 0.80 | 0.70 | 0.90 |
| Number of acts per partner | **N** | 70 | 70 | 70 | 70 | 70 | 70 |
| Number of partners | **M** | 1.0 | 0.001 | 0.003 | 1.0 | 0.001 | 0.003 |
| Probability |  | 0.00032 | 0.00000 | 0.00000 | 0.00076 | 0.00000 | 0.00000 |
| Total per 1000 |  | 0.32 | 0.000 | 0.0000 | 0.76 | 0.00 | 0.001 |
| Proportion already infected | **I** | 0.01 | 0.01 | 0.01 | 0.01 | 0.01 | 0.01 |
| Total per 1000 - I adjusted |  | 0.32 | 0.000 | 0.00000 | 0.76 | 0.00 | 0.001 |
|  |  |  |  | **0.320** |  |  | **0.761** |

**Impact of IEC intervention on risk behaviour**¶

Reduction in condom non-use: 2%

Reduction in partners: 0%

Reduction in STI non-treatment: 0%

**Annual number of new HIV infection averted among 1,000 men receiving IEC intervention = 0.02**

(Infections occurring in base scenario minus infection occurring with intervention)

**Women**

**Annual new HIV infections – base scenario without intervention**

|  | | **Risk of acquiring HIV** | | | **Risk of transmitting HIV** | | |
| --- | --- | --- | --- | --- | --- | --- | --- |
|  |  | **Receptive vaginal** | **Receptive anal** | **Receptive oral** | **Insertive vaginal** | **Insertive anal** | **Insertive oral** |
| HIV prevalence in sex partners* | **P** | 0.012 | 0.012 | 0.012 | 0.010 | 0.010 | 0.010 |
| Risk of HIV per unprotected act† | **R** | 0.0010 | 0.0073 | 0.0003 | 0.0005 | 0.0007 | 0.0000 |
| Fraction of acts with condom‡ | **F** | 0.08 | 0.08 | 0.00 | 0.08 | 0.08 | 0.00 |
| Effectiveness of condom§ | **E** | 0.80 | 0.70 | 0.90 | 0.80 | 0.70 | 0.90 |
| Number of acts per partner‡ | **N** | 70 | 70 | 70 | 70 | 70 | 70 |
| Number of partners‡ | **M** | 0.8 | 0.001 | 0.002 | 0.80 | 0.001 | 0.002 |
| Probability |  | 0.00062 | 0.00000 | 0.00000 | 0.00026 | 0.00000 | 0.00000 |
| Total per 1000 |  | 0.62 | 0.00 | 0.000 | 0.26 | 0.000 | 0.0000 |
| Proportion already infected | **I** | 0.01 | 0.01 | 0.01 | 0.01 | 0.01 | 0.01 |
| Total per 1000 - I adjusted |  | 0.61 | 0.00 | 0.00 | 0.26 | 0.000 | 0.0000 |
|  |  |  |  | **0.618** |  |  | **0.260** |

**Annual new HIV infections with IEC intervention**

|  | | **Risk of acquiring HIV** | | | **Risk of transmitting HIV** | | |
| --- | --- | --- | --- | --- | --- | --- | --- |
|  |  | **Receptive vaginal** | **Receptive anal** | **Receptive oral** | **Insertive vaginal** | **Insertive anal** | **Insertive oral** |
| HIV prevalence in sex partners | **P** | 0.012 | 0.012 | 0.012 | 0.010 | 0.010 | 0.010 |
| Risk of HIV per unprotected act | **R** | 0.0010 | 0.0073 | 0.0003 | 0.0005 | 0.0007 | 0.0000 |
| Fraction of acts with condom¶ | **F** | 0.10 | 0.098 | 0 | 0.098 | 0.098 | 0 |
| Effectiveness of condom | **E** | 0.80 | 0.70 | 0.90 | 0.80 | 0.70 | 0.90 |
| Number of acts per partner | **N** | 70 | 70 | 70 | 70 | 70 | 70 |
| Number of partners | **M** | 0.8 | 0.001 | 0.002 | 0.800 | 0.0008 | 0.002 |
| Probability |  | 0.00061 | 0.00000 | 0.00000 | 0.00026 | 0.00000 | 0.00000 |
| Total per 1000 |  | 0.61 | 0.00 | 0.0005 | 0.26 | 0.000 | 0.0000 |
| Proportion already infected | **I** | 0.01 | 0.01 | 0.01 | 0.01 | 0.01 | 0.01 |
| Total per 1000 - I adjusted |  | 0.60 | 0.00 | 0.00 | 0.26 | 0.000 | 0.0000 |
|  |  |  |  | **0.609** |  |  | **0.256** |

**Impact of IEC intervention on risk behaviour**¶

Reduction in condom non-use: 2%

Reduction in partners: 0%

Reduction in STI non-treatment: 0%

**Annual number of new HIV infection averted among 1,000 women receiving intervention = 0.01**

(Infections occurring in base scenario minus infection occurring with intervention)

**Annual number of new HIV infection averted among 1,000 men and women receiving intervention (weighted equally for men and women in the population) = 0.02**

*HIV prevalence for men and women in general population 15 to 49 years old was based on the recent National Family Health Survey- 3 data which found 1.2% prevalence in men and 0.8% in women in Andhra Pradesh state, the prevalence for women rounded off to 1% (http://www.nfhsindia.org/NFHS-3%20Data/VOL-1/Chapter%2012%20-%20HIV%20Prevalence%20).

†Risk of HIV per unprotected sex act was based on estimates used by UNAIDS (Epidemiological software and tools, 2005; <http://www.unaids.org/en/HIV_data/Epidemiology/episoftware.asp>), or if some risk values were not available from this source they were adapted from published literature. The risk of transmission in receptive vaginal sex was considered two times higher than insertive vaginal sex. The risk of transmission in receptive anal sex was considered five times higher than receptive vaginal sex. The risk of transmission in insertive anal sex was considered 1.5 times higher than insertive vaginal sex. The risk of transmission in receptive oral sex was considered six times less than receptive vaginal sex. The risk of transmission in insertive oral sex was considered ten times less than receptive oral sex. The risk of transmission was considered three times higher with STI than without STI. We assumed that 2% of all sex acts in the general population were with STI, which is based on estimated local trends. The combined transmission probability of risky unprotected sex acts is therefore weighted for 2% acts with STI and 98% acts without STI in the base case scenario.

‡Fraction of acts with condom, number of acts per partner and number of partners for men and women in the general population in base scenario were adapted from data from a population-based study in Andhra Pradesh (Dandona et al, BMC Medicine 2006;4:31 and Dandona et al, International Journal of Epidemiology 2008;37:1274-86).

§Effectiveness of condom was estimated to be 80% for vaginal sex (Weller and Davis, Cochrane Database Syst Rev 2002;1:CD003255). We assumed effectiveness of condom for anal sex to be 70% and for oral sex to be 90% (Dandona et al, BMC Public Health 2006;6:31).

¶Intervention impact on reduction in condom non-use was based on estimated local trends.

**Condom promotion programme**

**Men**

**Annual new HIV infections – base scenario without intervention**

|  |  | **Risk of acquiring HIV** | | | **Risk of transmitting HIV** | | |
| --- | --- | --- | --- | --- | --- | --- | --- |
|  |  | **Insertive vaginal** | **Insertive anal** | **Insertive oral** | **Receptive vaginal** | **Receptive anal** | **Receptive oral** |
| HIV prevalence in sex partners* | **P** | 0.02 | 0.02 | 0.02 | 0.02 | 0.02 | 0.02 |
| Risk of HIV per unprotected act† | **R** | 0.0008 | 0.0011 | 0.0000 | 0.0015 | 0.0110 | 0.0004 |
| Fraction of acts with condom‡ | **F** | 0.15 | 0.15 | 0.00 | 0.15 | 0.15 | 0.00 |
| Effectiveness of condom§ | **E** | 0.80 | 0.70 | 0.90 | 0.80 | 0.70 | 0.90 |
| Number of acts per partner‡ | **N** | 65 | 65 | 65 | 65 | 65 | 65 |
| Number of partners‡ | **M** | 1.5 | 0.002 | 0.004 | 1.5 | 0.002 | 0.004 |
| Probability |  | 0.00129 | 0.00000 | 0.00000 | 0.00253 | 0.00001 | 0.00000 |
| Total per 1000 |  | 1.29 | 0.002 | 0.0000 | 2.53 | 0.01 | 0.002 |
| Proportion already infected | **I** | 0.02 | 0.02 | 0.02 | 0.020 | 0.020 | 0.020 |
| Total per 1000 - I adjusted |  | 1.27 | 0.002 | 0.0000 | 2.48 | 0.01 | 0.002 |
|  |  |  |  | **1.27** |  |  | **2.50** |

**Annual new HIV infections with intervention**

| **General population Men** |  | **Risk of acquiring HIV** | | | **Risk of transmitting HIV** | | |
| --- | --- | --- | --- | --- | --- | --- | --- |
|  |  | **Insertive vaginal** | **Insertive anal** | **Insertive oral** | **Receptive vaginal** | **Receptive anal** | **Receptive oral** |
| HIV prevalence in sex partners | **P** | 0.02 | 0.02 | 0.02 | 0.02 | 0.02 | 0.02 |
| Risk of HIV per unprotected act | **R** | 0.0008 | 0.0011 | 0.0000 | 0.0015 | 0.0110 | 0.0004 |
| Fraction of acts with condom¶ | **F** | 0.22 | 0.22 | 0.00 | 0.22 | 0.22 | 0.00 |
| Effectiveness of condom | **E** | 0.80 | 0.70 | 0.90 | 0.80 | 0.70 | 0.90 |
| Number of acts per partner | **N** | 65 | 65 | 65 | 65 | 65 | 65 |
| Number of partners | **M** | 1.50 | 0.002 | 0.004 | 1.50 | 0.002 | 0.004 |
| Probability |  | 0.00121 | 0.00000 | 0.00000 | 0.00238 | 0.00001 | 0.00000 |
| Total per 1000 |  | 1.21 | 0.002 | 0.0000 | 2.38 | 0.01 | 0.002 |
| Proportion already infected | **I** | 0.02 | 0.02 | 0.02 | 0.02 | 0.02 | 0.02 |
| Total per 1000 - I adjusted |  | 1.19 | 0.002 | 0.0000 | 2.33 | 0.01 | 0.002 |
|  |  |  |  | **1.19** |  |  | **2.35** |

**Impact of intervention on risk behaviour**¶

Reduction in condom non-use: 8%

Reduction in partners: 0%

Reduction in STI non-treatment: 0%

**Annual number of new HIV infection averted among 1,000 men receiving intervention = 0.22**

(Infections occurring in base scenario minus infection occurring with intervention)

**Women**

**Annual new HIV infections – base scenario without intervention**

|  |  | **Risk of acquiring HIV** | | | **Risk of transmitting HIV** | | |
| --- | --- | --- | --- | --- | --- | --- | --- |
|  |  | **Receptive vaginal** | **Receptive anal** | **Receptive oral** | **Insertive vaginal** | **Insertive anal** | **Insertive oral** |
| HIV prevalence in sex partners* | **P** | 0.02 | 0.02 | 0.02 | 0.02 | 0.02 | 0.02 |
| Risk of HIV per unprotected act† | **R** | 0.0015 | 0.0110 | 0.0004 | 0.0008 | 0.0011 | 0.0000 |
| Fraction of acts with condom‡ | **F** | 0.15 | 0.15 | 0 | 0.15 | 0.15 | 0 |
| Effectiveness of condom§ | **E** | 0.80 | 0.70 | 0.90 | 0.80 | 0.70 | 0.90 |
| Number of acts per partner‡ | **N** | 65 | 65 | 65 | 65 | 65 | 65 |
| Number of partners‡ | **M** | 1.5 | 0.002 | 0.004 | 1.50 | 0.00 | 0.004 |
| Probability |  | 0.00253 | 0.00001 | 0.00000 | 0.00129 | 0.00000 | 0.00000 |
| Total per 1000 |  | 2.53 | 0.01 | 0.002 | 1.29 | 0.002 | 0.0000 |
| Proportion already infected | **I** | 0.02 | 0.02 | 0.02 | 0.02 | 0.02 | 0.02 |
| Total per 1000 - I adjusted |  | 2.48 | 0.01 | 0.002 | 1.27 | 0.002 | 0.0000 |
|  |  |  |  | **2.50** |  |  | **1.27** |

**Annual new HIV infections with intervention**

|  |  | **Risk of acquiring HIV** | | | **Risk of transmitting HIV** | | |
| --- | --- | --- | --- | --- | --- | --- | --- |
|  |  | **Receptive vaginal** | **Receptive anal** | **Receptive oral** | **Insertive vaginal** | **Insertive anal** | **Insertive oral** |
| HIV prevalence in sex partners | **P** | 0.02 | 0.02 | 0.02 | 0.02 | 0.02 | 0.02 |
| Risk of HIV per unprotected act | **R** | 0.0015 | 0.0110 | 0.0004 | 0.0008 | 0.0011 | 0.0000 |
| Fraction of acts with condom¶ | **F** | 0.19 | 0.19 | 0.00 | 0.19 | 0.19 | 0.00 |
| Effectiveness of condom | **E** | 0.80 | 0.70 | 0.90 | 0.80 | 0.70 | 0.90 |
| Number of acts per partner | **N** | 65 | 65 | 65 | 65 | 65 | 65 |
| Number of partners | **M** | 1.50 | 0.002 | 0.004 | 1.50 | 0.0015 | 0.004 |
| Probability |  | 0.00244 | 0.00001 | 0.00000 | 0.00124 | 0.00000 | 0.00000 |
| Total per 1000 |  | 2.44 | 0.01 | 0.002 | 1.24 | 0.001 | 0.0000 |
| Proportion already infected | **I** | 0.02 | 0.02 | 0.02 | 0.02 | 0.02 | 0.02 |
| Total per 1000 - I adjusted |  | 2.39 | 0.01 | 0.002 | 1.22 | 0.001 | 0.0000 |
|  |  |  |  | **2.40** |  |  | **1.22** |

**Impact of intervention on risk behaviour**¶

Reduction in condom non-use: 5%

Reduction in partners: 0%

Reduction in STI non-treatment: 0%

**Annual number of new HIV infection averted among 1,000 women receiving intervention = 0.14**

(Infections occurring in base scenario minus infection occurring with intervention)

**Annual number of new HIV infections averted among 1,000 men and women receiving intervention (weighted for men 0.75 and women 0.25)** ׀׀**= 0.20**

*HIV prevalence in men and women covered by the condom promotion programme was assumed based on programme data and estimated local trends.

†Risk of HIV per unprotected sex act was based on estimates used by UNAIDS (Epidemiological software and tools, 2005; <http://www.unaids.org/en/HIV_data/Epidemiology/episoftware.asp>), or if some risk values were not available from this source they were adapted from published literature. The risk of transmission in receptive vaginal sex was considered two times higher than insertive vaginal sex. The risk of transmission in receptive anal sex was considered five times higher than receptive vaginal sex. The risk of transmission in insertive anal sex was considered 1.5 times higher than insertive vaginal sex. The risk of transmission in receptive oral sex was considered six times less than receptive vaginal sex. The risk of transmission in insertive oral sex was considered ten times less than receptive oral sex. The risk of transmission was considered three times higher with STI than without STI. We assumed that 5% of all sex acts in the population covered by this programme were with STI, which is based on estimates from unpublished local data/trends. The combined transmission probability of risky unprotected sex acts is therefore weighted for 5% acts with STI and 95% acts without STI in the base scenario.

‡Fraction of acts with condom, number of acts per partner and number of partners for men and women in base scenario were adapted from data from a population-based study in Andhra Pradesh (Dandona et al, BMC Medicine 2006;4:31 and Dandona et al, International Journal of Epidemiology 2008;37:1274-86).

§Effectiveness of condom was estimated to be 80% for vaginal sex (Weller and Davis, Cochrane Database Syst Rev 2002;1:CD003255). We assumed effectiveness of condom for anal sex to be 70% and for oral sex to be 90% (Dandona et al, BMC Public Health 2006;6:31).

¶Intervention impact on reduction in condom non-use was based on estimated local trends.

׀׀Proportion of men and women covered by condom promotion intervention from programme data.

**Prevention of parent to child transmission (PPTCT) clinics**

In order to calculate the annual number of new HIV infections averted among every 1,000 pregnant women receiving PPTCT, we used data from our sample on the number of pregnant women receiving PPTCT, number of pregnant women found HIV positive, number of medical termination of pregnancies, number of HIV positive deliveries, and number of mother-neonate pairs received nevirapine. We assumed the vertical transmission rate of HIV without treatment to be 25% and the efficacy of nevirapine to prevent transmission to be 40% based on literature (WHO, Guidelines on care, treatment and support for women living with HIV/AIDS and their children in resource-constrained settings, 2004).

**Blood banks**

In order to calculate the annual number of new HIV infections averted among every 1,000 blood units screened by blood banks we used data from our sample on the number of units screened and number found HIV positive. We assumed 92% efficacy of HIV transmission from transfusion of infected blood based on literature (Baggaley et al, AIDS 2006;20:805-12).

**Assumptions used for intervention effectiveness sensitivity analysis**

**Voluntary counselling and testing (VCT) centres**

The plausible ranges considered for the variables were as follows:

| **Variable** | **Plausible range** |
| --- | --- |
| ***Probability of HIV infection*** | |
| HIV prevalence in sex partners [P] | 20% lower and higher than the point estimate for P |
| Risk of HIV per unprotected act [R] | 20% lower and higher than the point estimate for R |
| Fraction of acts with condom [F] | 20% lower and higher than the point estimate for F |
| Effectiveness of condom [E] | 0.75–0.85 for vaginal sex, 0.65–0.75 for anal sex, and 0.85–0.95 for oral sex (Dandona et al, BMC Public Health 2006;6:31) |
| Number of acts per partner [N] | 20% lower and higher than the point estimate for N |
| Number of partners [M] | 20% lower and higher than the point estimate for M |
| Proportion already infected [I] | 20% lower and higher than the point estimate for I |
| ***Intervention impact*** | |
| Reduction in condom non-use | Low and high values for reduction in condom non-use with average VCTC intervention impact from Stover et al (Science 2006;311:1474-76) |

**Sexually transmitted infection (STI) clinics**

The plausible ranges considered for the variables were as follows:

| **Variable** | **Plausible range** |
| --- | --- |
| ***Probability of HIV infection*** | |
| HIV prevalence in sex partners [P] | 20% lower and higher than the point estimate for P |
| Risk of HIV per unprotected act [R] | 20% lower and higher than the point estimate for R |
| Fraction of acts with condom [F] | 20% lower and higher than the point estimate for F |
| Effectiveness of condom [E] | 0.75–0.85 for vaginal sex, 0.65–0.75 for anal sex, and 0.85–0.95 for oral sex (Dandona et al, BMC Public Health 2006;6:31) |
| Number of acts per partner [N] | 20% lower and higher than the point estimate for N |
| Number of partners [M] | 20% lower and higher than the point estimate for M |
| Proportion already infected [I] | 20% lower and higher than the point estimate for I |
| ***Intervention impact*** | |
| Reduction in condom non-use | Half the low and high values for reduction in condom non-use with average VCTC intervention impact from Stover et al (Science 2006;311:1474-76) |
| Reduction in STI non-treatment | 20% lower and higher than the estimate for reduction in STI non-treatment |

**Prevention of parent to child transmission (PPTCT) clinics**

The plausible ranges considered for the variables were as follows:

| **Variable** | **Plausible range** |
| --- | --- |
| Proportion of HIV positive pregnant women received test results to tested HIV positive | 20% lower and higher than the proportion |
| Proportion of MTPs to HIV+ pregnant women received post test counseled/test results | 20% lower and higher than the proportion |
| Proportion of post test counseled to tested for pregnant women | 5% lower and higher than the proportion |
| Transmission Rate | 20% lower and higher than the estimated transmission rate |
| Efficacy of Nevirapine | 20% lower and higher than the efficacy of Nevirapine |

**Blood banks**

The plausible ranges considered for the variables were as follows:

| **variable** | **Plausible range** |
| --- | --- |
| Proportion of units of blood tested positive | 20% lower and higher than the proportion |
| Efficiency of units of blood tested positive | 0.88–0.96 for efficiency of units of blood tested HIV positive from Baggaley et al (AIDS 2006;20:805-12) |

**Women sex worker (SW) programmes**

**Women sex workers**

**The plausible ranges considered for the variables were as follows:**

| **Variable** | **Plausible range** |
| --- | --- |
| ***Probability of HIV infection*** | |
| HIV prevalence in sex partners [P] | 20% lower and higher than the point estimate for P |
| Risk of HIV per unprotected act [R] | 20% lower and higher than the point estimate for R |
| Fraction of acts with condom [F] | 20% lower and higher than the point estimate for F |
| Effectiveness of condom [E] | 0.75–0.85 for vaginal sex, 0.65–0.75 for anal sex, and 0.85–0.95 for oral sex (Dandona et al, BMC Public Health 2006;6:31) |
| Number of acts per partner [N] | 20% lower and higher than the point estimate for N |
| Number of partners [M] | low and high values of the 95% confidence interval for the point estimate for M from Dandona et al (BMC Public Health 2005;5:87) |
| Proportion already infected [I] | 20% lower and higher than the point estimate for I |
| ***Intervention impact*** | |
| Reduction in condom non-use | High values for reduction in condom non-use with low and high sex worker intervention impact from Stover et al (Science 2006;311:1474-76) |
| Reduction in partners | Lower value assumed as 0 and higher value as the higher value for reduction in partners with high sex worker intervention impact from Stover et al (Science 2006;311:1474-76) |
| Reduction in STI non-treatment | 20% lower and higher than the estimate for reduction in STI non-treatment |

**Other women sex partners of clients of women sex workers**

**The plausible ranges considered for the variables were as follows:**

| **Variable** | **Plausible range** |
| --- | --- |
| ***Probability of HIV infection*** | |
| HIV prevalence in sex partners [P] | 20% lower and higher than the point estimate for P |
| Risk of HIV per unprotected act [R] | 20% lower and higher than the point estimate for R |
| Fraction of acts with condom [F] | 20% lower and higher than the point estimate for F |
| Effectiveness of condom [E] | 0.75–0.85 for vaginal sex, 0.65–0.75 for anal sex, and 0.85–0.95 for oral sex (Dandona et al, BMC Public Health 2006;6:31) |
| Number of acts per partner [N] | 20% lower and higher than the point estimate for N |
| Number of partners [M] | 20% lower and higher than the point estimate for M |
| Proportion already infected [I] | 20% lower and higher than the point estimate for I |
| ***Intervention impact*** | |
| Reduction in condom non-use | 20% lower and higher than the estimate for reduction in condom non-use |

**Men who have sex with men (MSM) programmes**

**The plausible ranges considered for the variables were as follows:**

| **Variable** | **Plausible range** |
| --- | --- |
| ***Probability of HIV infection*** | |
| HIV prevalence in sex partners [P] | 20% lower and higher than the point estimate for P |
| Risk of HIV per unprotected act [R] | 20% lower and higher than the point estimate for R |
| Fraction of acts with condom [F] | 20% lower and higher than the point estimate for F |
| Effectiveness of condom [E] | 0.75–0.85 for vaginal sex, 0.65–0.75 for anal sex, and 0.85–0.95 for oral sex (Dandona et al, BMC Public Health 2006;6:31) |
| Number of acts per partner [N] | 20% lower and higher than the point estimate for N |
| Number of partners [M] | 20% lower and higher than the point estimate for M |
| Proportion already infected [I] | 20% lower and higher than the point estimate for I |
| ***Intervention impact*** | |
| Reduction in condom non-use | For sex with men, high values for reduction in condom non-use with low and high MSM intervention impacts from Stover et al (Science 2006;311:1474-76) and for MSM sex with women, 20% lower and higher than the estimates for reduction in condom non-use |
| Reduction in partners | 20% lower and higher than the estimate for reduction in partners |
| Reduction in STI non-treatment | 20% lower and higher than the estimate for reduction in STI non-treatment |

**Trucker programmes**

The plausible ranges considered for the variables were as follows:

| **Variable** | **Plausible range** |
| --- | --- |
| ***Probability of HIV infection*** | |
| HIV prevalence in sex partners [P] | 20% lower and higher than the point estimate for P |
| Risk of HIV per unprotected act [R] | 20% lower and higher than the point estimate for R |
| Fraction of acts with condom [F] | 20% lower and higher than the point estimate for F |
| Effectiveness of condom [E] | 0.75–0.85 for vaginal sex, 0.65–0.75 for anal sex, and 0.85–0.95 for oral sex (Dandona et al, BMC Public Health 2006;6:31) |
| Number of acts per partner [N] | 95% confidence interval for the point estimate for N from Dandona et al (BMC Medicine 2006;4:31) |
| Number of partners [M] | 20% lower and higher than the point estimate for M |
| Proportion already infected [I] | 20% lower and higher than the point estimate for I |
| ***Intervention impact*** | |
| Reduction in condom non-use | Half of high values for reduction in condom non-use with low and high sex worker intervention impact from Stover et al (Science 2006;311:1474-76) |
| Reduction in STI non-treatment | 20% lower and higher than the estimate for reduction in STI non-treatment |

**Composite programmes**

The plausible ranges considered for the variables were as follows:

| **Variable** | **Plausible range** |
| --- | --- |
| ***Probability of HIV infection*** | |
| HIV prevalence in sex partners [P] | 20% lower and higher than the point estimate for P |
| Risk of HIV per unprotected act [R] | 20% lower and higher than the point estimate for R |
| Fraction of acts with condom [F] | 20% lower and higher than the point estimate for F |
| Effectiveness of condom [E] | 0.75–0.85 for vaginal sex, 0.65–0.75 for anal sex, and 0.85–0.95 for oral sex (Dandona et al, BMC Public Health 2006;6:31) |
| Number of acts per partner [N] | 20% lower and higher than the point estimate for N |
| Number of partners [M] | 20% lower and higher than the point estimate for M |
| Proportion already infected [I] | 20% lower and higher than the point estimate for I |
| ***Intervention impact*** | |
| Reduction in condom non-use | 20% lower and higher than the estimate for reduction in condom non-use |
| Reduction in partners | 20% lower and higher than the estimate for reduction in partners |
| Reduction in STI non-treatment | 20% lower and higher than the estimate for reduction in STI non-treatment |

**Workplace programmes**

The plausible ranges considered for the variables were as follows:

| **Variable** | **Plausible range** |
| --- | --- |
| ***Probability of HIV infection*** | |
| HIV prevalence in sex partners [P] | 95% confidence interval for the point estimate for P from Dandona et al (BMC Medicine 2006;4:31) |
| Risk of HIV per unprotected act [R] | 20% lower and higher than the point estimate for R |
| Fraction of acts with condom [F] | 20% lower and higher than the point estimate for F |
| Effectiveness of condom [E] | 0.75–0.85 for vaginal sex, 0.65–0.75 for anal sex, and 0.85–0.95 for oral sex (Dandona et al, BMC Public Health 2006;6:31) |
| Number of acts per partner [N] | 20% lower and higher than the point estimate for N |
| Number of partners [M] | 20% lower and higher than the point estimate for M |
| Proportion already infected [I] | 95% confidence interval for the point estimate for I from Dandona et al (BMC Medicine 2006;4:31) |
| ***Intervention impact*** | |
| Reduction in condom non-use | Low and high values for reduction in condom non-use with average workplace intervention impact from Stover et al (Science 2006;311:1474-76) |
| Reduction in partners | 20% lower and higher than the estimate for reduction in partners |
| Reduction in STI non-treatment | 20% lower and higher than the estimate for reduction in STI non-treatment |

**Migrant labourer programmes**

The plausible ranges considered for the variables were as follows:

| **Variable** | **Plausible range** |
| --- | --- |
| ***Probability of HIV infection*** | |
| HIV prevalence in sex partners [P] | For insertive sex, 20% lower and higher than the point estimate for P and 95% confidence interval for the point estimate for P for receptive sex from Dandona et al (BMC Medicine 2006;4:31) |
| Risk of HIV per unprotected act [R] | 20% lower and higher than the point estimate for R |
| Fraction of acts with condom [F] | 20% lower and higher than the point estimate for F |
| Effectiveness of condom [E] | 0.75–0.85 for vaginal sex, 0.65–0.75 for anal sex, and 0.85–0.95 for oral sex (Dandona et al, BMC Public Health 2006;6:31) |
| Number of acts per partner [N] | 95% confidence interval for the point estimate for N from Dandona et al (BMC Medicine 2006;4:31) |
| Number of partners [M] | 20% lower and higher than the point estimate for M |
| Proportion already infected [I] | For receptive sex, 20% lower and higher than the point estimate for I and 95% confidence interval for the point estimate for I for insertive sex from Dandona et al (BMC Medicine 2006;4:31) |
| ***Intervention impact*** | |
| Reduction in condom non-use | Low and high values for reduction in condom non-use with average workplace intervention impact from Stover et al (Science 2006;311:1474-76) |
| Reduction in partners | 20% lower and higher than the estimate for reduction in partners |
| Reduction in STI non-treatment | 20% lower and higher than the estimate for reduction in STI non-treatment |

**Street children programmes**

The plausible ranges considered for the variables were as follows:

| **Variable** | **Plausible range** |
| --- | --- |
| ***Probability of HIV infection*** | |
| HIV prevalence in sex partners [P] | 20% lower and higher than the point estimate for P |
| Risk of HIV per unprotected act [R] | 20% lower and higher than the point estimate for R |
| Fraction of acts with condom [F] | 20% lower and higher than the point estimate for F |
| Effectiveness of condom [E] | 0.75–0.85 for vaginal sex, 0.65–0.75 for anal sex, and 0.85–0.95 for oral sex (Dandona et al, BMC Public Health 2006;6:31) |
| Number of acts per partner [N] | 20% lower and higher than the point estimate for N |
| Number of partners [M] | 20% lower and higher than the point estimate for M |
| Proportion already infected [I] | 20% lower and higher than the point estimate for I |
| ***Intervention impact*** | |
| Reduction in condom non-use | 20% lower and higher than the estimate for reduction in condom non-use |
| Reduction in partners | 20% lower and higher than the estimate for reduction in partners |
| Reduction in STI non-treatment | 20% lower and higher than the estimate for reduction in STI non-treatment |

**Prisoner programmes**

The plausible ranges considered for the variables were as follows:

| **Variable** | **Plausible range** |
| --- | --- |
| ***Probability of HIV infection*** | |
| HIV prevalence in sex partners [P] | 20% lower and higher than the point estimate for P |
| Risk of HIV per unprotected act [R] | 20% lower and higher than the point estimate for R |
| Fraction of acts with condom [F] | 20% lower and higher than the point estimate for F |
| Effectiveness of condom [E] | 0.75–0.85 for vaginal sex, 0.65–0.75 for anal sex, and 0.85–0.95 for oral sex (Dandona et al, BMC Public Health 2006;6:31) |
| Number of acts per partner [N] | 20% lower and higher than the point estimate for N |
| Number of partners [M] | 20% lower and higher than the point estimate for M |
| Proportion already infected [I] | 20% lower and higher than the point estimate for I |
| ***Intervention impact*** | |
| Reduction in condom non-use | 20% lower and higher than the estimate for reduction in condom non-use |
| Reduction in STI non-treatment | 20% lower and higher than the estimate for reduction in STI non-treatment |

**IEC for general public**

The plausible ranges considered for the variables were as follows:

| **Variable** | **Plausible range** |
| --- | --- |
| ***Probability of HIV infection*** | |
| HIV prevalence in sex partners [P] | 20% lower and higher than the point estimate for P |
| Risk of HIV per unprotected act [R] | 20% lower and higher than the point estimate for R |
| Fraction of acts with condom [F] | 95% confidence interval for the point estimate for F from Dandona et al (BMC Medicine 2006;4:31) |
| Effectiveness of condom [E] | 0.75–0.85 for vaginal sex, 0.65–0.75 for anal sex, and 0.85–0.95 for oral sex (Dandona et al, BMC Public Health 2006;6:31) |
| Number of acts per partner [N] | 20% lower and higher than the point estimate for N |
| Number of partners [M] | 95% confidence interval for the point estimate for M from Dandona et al (BMC Medicine 2006;4:31) |
| Proportion already infected [I] | 20% lower and higher than the point estimate for I |
| ***Intervention impact*** | |
| Reduction in condom non-use | 20% lower and higher than the estimate for reduction in condom non-use |

**Condom Promotion Programme (CPP)**

The plausible ranges considered for the variables were as follows:

| **Variable** | **Plausible range** |
| --- | --- |
| ***Probability of HIV infection*** | |
| HIV prevalence in sex partners [P] | 20% lower and higher than the point estimate for P |
| Risk of HIV per unprotected act [R] | 20% lower and higher than the point estimate for R |
| Fraction of acts with condom [F] | 95% confidence interval for the point estimate for F from Dandona et al (BMC Medicine 2006;4:31) |
| Effectiveness of condom [E] | 0.75–0.85 for vaginal sex, 0.65–0.75 for anal sex, and 0.85–0.95 for oral sex (Dandona et al, BMC Public Health 2006;6:31) |
| Number of acts per partner [N] | 20% lower and higher than the point estimate for N |
| Number of partners [M] | 95% confidence interval for the point estimate for M from Dandona et al (BMC Medicine 2006;4:31) |
| Proportion already infected [I] | 20% lower and higher than the point estimate for I |
| ***Intervention impact*** | |
| Reduction in condom non-use | 20% lower and higher than the estimate for reduction in condom non-use |
