# Supplementary material for: Cost-effectiveness of HIV prevention interventions in Andhra Pradesh state of India
Source: BMC Health Serv Res. 2010 May 10;10:117. doi: 10.1186/1472-6963-10-117 (PMC2874552; doi:10.1186/1472-6963-10-117)
Supplement: Additional file 2 — Calculation of Disability Adjusted Life Years (DALYs) saved. This file shows calculation of Disability Adjusted Life Years (DALYs) saved for each HIV infection averted by HIV prevention interventions in Andhra Pradesh state of India. [file 1472-6963-10-117-S2.DOC]

**Cost-effectiveness of HIV prevention interventions in Andhra Pradesh state of India**

Lalit Dandona, S G Prem Kumar, G Anil Kumar, Rakhi Dandona

**Additional file:** Calculation of Disability Adjusted Life Years (DALYs) saved.

This file shows calculation of Disability Adjusted Life Years (DALYs) saved for each HIV infection averted by HIV prevention interventions in Andhra Pradesh state of India.

**Sexually acquired HIV**

**Calculation of DALYs saved per infection averted in adults (with age-weighting)**

***Men***

| **YLL** | | | **YLDs** | | | |
| --- | --- | --- | --- | --- | --- | --- |
| **Variables** | |  | **Variables** | | **Pre AIDS** | **AIDS** |
| Age weighting modulation factor | K | 1 | Age weighting modulation factor | K | 1 | 1 |
| Age weighting constant | C | 0.1658 | Age weighting constant | C | 0.1658 | 0.1658 |
| Discount rate | r | 0.03 | Discount rate | r | 0.03 | 0.03 |
| Age at death | a | 39.5 | Age of onset of the disability | a | 36 | 38 |
| Constant from the age weighting function | β | 0.04 | Constant from the age weighting function | β | 0.04 | 0.04 |
| Standard expectation of life at age | L | 41.16 | Duration of disability | L | 2 | 1.5 |
|  | e | 2.72 |  | e | 2.72 | 2.72 |
|  |  |  | Disability weight | D | 0.14 | 0.51 |
| YLL |  | 23.64 | YLDs |  | 0.37 | 1.01 |
| DALYs saved (YLL + YLDs) |  | 25.02 |  |  |  |  |

***Women***

| **YLL** | | | **YLDs** | | | |
| --- | --- | --- | --- | --- | --- | --- |
| **Variables** | |  | **Variables** | | **Pre AIDS** | **AIDS** |
| Age weighting modulation factor | K | 1 | Age weighting modulation factor | K | 1 | 1 |
| Age weighting constant | C | 0.1658 | Age weighting constant | C | 0.1658 | 0.1658 |
| Discount rate | r | 0.03 | Discount rate | r | 0.03 | 0.03 |
| Age at death | a | 34.5 | Age of onset of the disability | a | 31 | 33 |
| Constant from the age weighting function | β | 0.04 | Constant from the age weighting function | β | 0.04 | 0.04 |
| Standard expectation of life at age | L | 48.83 | Duration of disability | L | 2 | 1.5 |
|  | e | 2.72 |  | e | 2.72 | 2.72 |
|  |  |  | Disability weight | D | 0.14 | 0.51 |
| YLL |  | 27.16 | YLDs |  | 0.39 | 1.07 |
| DALYs saved (YLL + YLDs) |  | 28.63 |  |  |  |  |

**Calculation of DALYs saved per infection averted in adults (without age-weighting)**

***Men***

| **YLL** | | | **YLDs** | | | |
| --- | --- | --- | --- | --- | --- | --- |
| **Variables** | |  | **Variables** | | **Pre- AIDS** | **AIDS** |
| Age weighting modulation factor | K | 0 | Age weighting modulation factor | K | 0 | 0 |
| Age weighting constant | C | NA | Age weighting constant | C | NA | NA |
| Discount rate | r | 0.03 | Discount rate | r | 0.03 | 0.03 |
| Age at death | a | 39.5 | Age of onset of the disability | a | 36 | 38 |
| Constant from the age weighting function | β | NA | Constant from the age weighting function | β | NA | NA |
| Standard expectation of life at age | L | 41.16 | Duration of disability | L | 2 | 1.5 |
|  | e | 2.72 |  | e | 2.72 | 2.72 |
|  |  |  | Disability weight | D | 0.14 | 0.51 |
| YLL |  | 23.64 | YLDs |  | 0.26 | 0.74 |
| DALYs saved (YLL + YLDs) |  | 24.64 |  |  |  |  |

***Women***

| **YLL** | | | **YLDs** | | | |
| --- | --- | --- | --- | --- | --- | --- |
| **Variables** | |  | **Variables** | | **Pre- AIDS** | **AIDS** |
| Age weighting modulation factor | K | 0 | Age weighting modulation factor | K | 0 | 0 |
| Age weighting constant | C | NA | Age weighting constant | C | NA | NA |
| Discount rate | r | 0.03 | Discount rate | r | 0.03 | 0.03 |
| Age at death | a | 34.5 | Age of onset of the disability | a | 31 | 33 |
| Constant from the age weighting function | β | NA | Constant from the age weighting function | β | NA | NA |
| Standard expectation of life at age | L | 48.83 | Duration of disability | L | 2 | 1.5 |
|  | e | 2.72 |  | e | 2.72 | 2.72 |
|  |  |  | Disability weight | D | 0.14 | 0.51 |
| YLL |  | 25.63 | YLDs |  | 0.26 | 0.74 |
| DALYs saved (YLL + YLDs) |  | 26.64 |  |  |  |  |

NA means not applicable

**DALYs saved by each intervention preventing sexual acquisition of HIV**

Average DALYs saved per HIV infection averted by each intervention weighted for the proportion of infections averted in men and women based on the services provided to each gender, using the above calculations of DALYs saved for sexually acquired HIV.

**Voluntary counselling and testing (VCT) centres**

DALYs saved (with age-weighting) = 28.1

DALYs saved (without age-weighting) = 26.3

**Sexually transmitted infection (STI) clinics**

DALYs saved (with age-weighting) = 28.0

DALYs saved (without age-weighting) = 26.3

**Women sex worker (SW) programmes**

DALYs saved (with age-weighting) = 27.8

DALYs saved (without age-weighting) = 26.2

**Men who have sex with men (MSM) programmes**

DALYs saved (with age-weighting) = 25.3

DALYs saved (without age-weighting) = 24.8

**Trucker programmes**

DALYs saved (with age-weighting) = 27.4

DALYs saved (without age-weighting) = 26.0

**Composite programmes**

DALYs saved (with age-weighting) = 27.3

DALYs saved (without age-weighting) = 25.9

**Workplace programmes**

DALYs saved (with age-weighting) = 27.4

DALYs saved (without age-weighting) = 26.0

**Migrant labourer programmes**

DALYs saved (with age-weighting) = 27.4

DALYs saved (without age-weighting) = 25.9

**Prisoner programmes**

DALYs saved (with age-weighting) = 27.4

DALYs saved (without age-weighting) = 26.0

**IEC for general public**

DALYs saved (with age-weighting) = 27.6

DALYs saved (without age-weighting) = 26.1

**Condom promotion programme**

DALYs saved (with age-weighting) = 27.4

DALYs saved (without age-weighting) = 26.0

**Street children programmes**

For sexually acquired HIV in street children, the average age at infection was assumed as 17 years for both boys and girls based on estimated local trends.

**Calculation of DALYs saved per infection averted (with age-weighting)**

***Boys***

| **YLL** | | | **YLDs** | | | |
| --- | --- | --- | --- | --- | --- | --- |
| **Variables** | |  | **Variables** | | **Pre- AIDS** | **AIDS** |
| Age weighting modulation factor | K | 1 | Age weighting modulation factor | K | 1 | 1 |
| Age weighting constant | C | 0.1658 | Age weighting constant | C | 0.1658 | 0.1658 |
| Discount rate | r | 0.03 | Discount rate | r | 0.03 | 0.03 |
| Age at death | a | 26.5 | Age of onset of the disability | a | 23 | 25 |
| Constant from the age weighting function | β | 0.04 | Constant from the age weighting function | β | 0.04 | 0.04 |
| Standard expectation of life at age | L | 51.01 | Duration of disability | L | 2 | 1.5 |
|  | e | 2.72 |  | e | 2.72 | 2.72 |
|  |  |  | Disability weight | D | 0.14 | 0.51 |
| YLL |  | 31.35 | YLDs |  | 0.40 | 1.13 |
| DALYs saved (YLL + YLDs) |  | 32.88 |  |  |  |  |

***Girls***

| **YLL** | | | **YLDs** | | | |
| --- | --- | --- | --- | --- | --- | --- |
| **Variables** | |  | **Variables** | | **Pre- AIDS** | **AIDS** |
| Age weighting modulation factor | K | 1 | Age weighting modulation factor | K | 1 | 1 |
| Age weighting constant | C | 0.1658 | Age weighting constant | C | 0.1658 | 0.1658 |
| Discount rate | r | 0.03 | Discount rate | r | 0.03 | 0.03 |
| Age at death | a | 26.5 | Age of onset of the disability | a | 23 | 25 |
| Constant from the age weighting function | β | 0.04 | Constant from the age weighting function | β | 0.04 | 0.04 |
| Standard expectation of life at age | L | 53.76 | Duration of disability | L | 2 | 1.5 |
|  | e | 2.72 |  | e | 2.72 | 2.72 |
|  |  |  | Disability weight | D | 0.14 | 0.51 |
| YLL |  | 31.67 | YLDs |  | 0.40 | 1.13 |
| DALYs saved (YLL + YLDs) |  | 33.20 |  |  |  |  |

DALYs saved (weighted average based on proportion of HIV infections averted in boys and girls) = 33.1

**Calculation of DALYs saved per infection averted (without age-weighting)**

***Boys***

| **YLL** | | | **YLDs** | | | |
| --- | --- | --- | --- | --- | --- | --- |
| **Variables** | |  | **Variables** | | **Pre- AIDS** | **AIDS** |
| Age weighting modulation factor | K | 0 | Age weighting modulation factor | K | 0 | 0 |
| Age weighting constant | C | NA | Age weighting constant | C | NA | NA |
| Discount rate | r | 0.03 | Discount rate | r | 0.03 | 0.03 |
| Age at death | a | 26.5 | Age of onset of the disability | a | 23 | 25 |
| Constant from the age weighting function | β | NA | Constant from the age weighting function | β | NA | NA |
| Standard expectation of life at age | L | 51.01 | Duration of disability | L | 2 | 1.5 |
|  | e | 2.72 |  | e | 2.72 | 2.72 |
|  |  |  | Disability weight | D | 0.14 | 0.51 |
| YLL |  | 26.12 | YLDs |  | 0.26 | 0.74 |
| DALYs saved (YLL + YLDs) |  | 27.12 |  |  |  |  |

***Girls***

| **YLL** | | | **YLDs** | | | |
| --- | --- | --- | --- | --- | --- | --- |
| **Variables** | |  | **Variables** | | **Pre- AIDS** | **AIDS** |
| Age weighting modulation factor | K | 0 | Age weighting modulation factor | K | 0 | 0 |
| Age weighting constant | C | NA | Age weighting constant | C | NA | NA |
| Discount rate | r | 0.03 | Discount rate | r | 0.03 | 0.03 |
| Age at death | a | 26.5 | Age of onset of the disability | a | 23 | 25 |
| Constant from the age weighting function | β | NA | Constant from the age weighting function | β | NA | NA |
| Standard expectation of life at age | L | 53.76 | Duration of disability | L | 2 | 1.5 |
|  | e | 2.72 |  | e | 2.72 | 2.72 |
|  |  |  | Disability weight | D | 0.14 | 0.51 |
| YLL |  | 26.69 | YLDs |  | 0.26 | 0.74 |
| DALYs saved (YLL + YLDs) |  | 27.69 |  |  |  |  |

NA means not applicable

DALYs saved (weighted average based on proportion of HIV infections averted in boys and girls) = 27.6

**Prevention of parent to child transmission (PPTCT) clinics**

The age at infection for mother to child transmission of HIV was considered as the first year of life.

**Calculation of DALYs saved per infection averted (with age-weighting)**

***Boys***

| **YLL** | | | **YLDs** | | | |
| --- | --- | --- | --- | --- | --- | --- |
| **Variables** | |  | **Variables** | | **Pre- AIDS** | **AIDS** |
| Age weighting modulation factor | K | 1 | Age weighting modulation factor | K | 1 | 1 |
| Age weighting constant | C | 0.1658 | Age weighting constant | C | 0.1658 | 0.1658 |
| Discount rate | r | 0.03 | Discount rate | r | 0.03 | 0.03 |
| Age at death | a | 11 | Age of onset of the disability | a | 7.5 | 9.5 |
| Constant from the age weighting function | β | 0.04 | Constant from the age weighting function | β | 0.04 | 0.04 |
| Standard expectation of life at age | L | 65.91 | Duration of disability | L | 2 | 1.5 |
|  | e | 2.72 |  | e | 2.72 | 2.72 |
|  |  |  | Disability weight | D | 0.12 | 0.51 |
| YLL |  | 37.19 | YLDs |  | 0.24 | 0.83 |
| DALYs saved (YLL + YLDs) |  | 38.27 |  |  |  |  |

***Girls***

| **YLL** | | | **YLDs** | | | |
| --- | --- | --- | --- | --- | --- | --- |
| **Variables** | |  | **Variables** | | **Pre- AIDS** | **AIDS** |
| Age weighting modulation factor | K | 1 | Age weighting modulation factor | K | 1 | 1 |
| Age weighting constant | C | 0.1658 | Age weighting constant | C | 0.1658 | 0.1658 |
| Discount rate | r | 0.03 | Discount rate | r | 0.03 | 0.03 |
| Age at death | a | 11 | Age of onset of the disability | a | 7.5 | 9.5 |
| Constant from the age weighting function | β | 0.04 | Constant from the age weighting function | β | 0.04 | 0.04 |
| Standard expectation of life at age | L | 68.52 | Duration of disability | L | 2 | 1.5 |
|  | e | 2.72 |  | e | 2.72 | 2.72 |
|  |  |  | Disability weight | D | 0.123 | 0.505 |
| YLL |  | 37.39 | YLDs |  | 0.24 | 0.83 |
| DALYs saved (YLL + YLDs) |  | 38.46 |  |  |  |  |

DALYs saved (weighted equally for boys and girls) = 38.4

**Calculation of DALYs saved per infection averted (without age-weighting)**

***Boys***

| **YLL** | | | **YLDs** | | | |
| --- | --- | --- | --- | --- | --- | --- |
| **Variables** | |  | **Variables** | | **Pre- AIDS** | **AIDS** |
| Age weighting modulation factor | K | 0 | Age weighting modulation factor | K | 0 | 0 |
| Age weighting constant | C | NA | Age weighting constant | C | NA | NA |
| Discount rate | r | 0.03 | Discount rate | r | 0.03 | 0.03 |
| Age at death | a | 11 | Age of onset of the disability | a | 7.5 | 9.5 |
| Constant from the age weighting function | β | NA | Constant from the age weighting function | β | NA | NA |
| Standard expectation of life at age | L | 65.91 | Duration of disability | L | 2 | 1.5 |
|  | e | 2.72 |  | e | 2.72 | 2.72 |
|  |  |  | Disability weight | D | 0.12 | 0.51 |
| YLL |  | 28.72 | YLDs |  | 0.24 | 0.74 |
| DALYs saved (YLL + YLDs) |  | 29.70 |  |  |  |  |

***Girls***

| **YLL** | | | **YLDs** | | | |
| --- | --- | --- | --- | --- | --- | --- |
| **Variables** | |  | **Variables** | | **Pre- AIDS** | **AIDS** |
| Age weighting modulation factor | K | 0 | Age weighting modulation factor | K | 0 | 0 |
| Age weighting constant | C | NA | Age weighting constant | C | NA | NA |
| Discount rate | r | 0.03 | Discount rate | r | 0.03 | 0.03 |
| Age at death | a | 11 | Age of onset of the disability | a | 7.5 | 9.5 |
| Constant from the age weighting function | β | NA | Constant from the age weighting function | β | NA | NA |
| Standard expectation of life at age | L | 68.52 | Duration of disability | L | 2 | 1.5 |
|  | e | 2.72 |  | e | 2.72 | 2.72 |
|  |  |  | Disability weight | D | 0.123 | 0.505 |
| YLL |  | 29.07 | YLDs |  | 0.24 | 0.74 |
| DALYs saved (YLL + YLDs) |  | 30.05 |  |  |  |  |

NA means not applicable

DALYs saved (weighted equally for boys and girls) = 29.9

**Blood banks**

The average age at infection from blood transfusion assumed as 5 years in the 14 years and less age group, 30 years in the 15-44 years age group, and 50 years in the 45 years and above age group, based on estimated local trends.

**Calculation of DALYs saved per infection averted (with age-weighting)**

***Boys age 14 years or less***

| **YLL** | | | **YLDs** | | | |
| --- | --- | --- | --- | --- | --- | --- |
| **Variables** | |  | **Variables** | | **Pre- AIDS** | **AIDS** |
| Age weighting modulation factor | K | 1 | Age weighting modulation factor | K | 1 | 1 |
| Age weighting constant | C | 0.1658 | Age weighting constant | C | 0.1658 | 0.1658 |
| Discount rate | r | 0.03 | Discount rate | r | 0.03 | 0.03 |
| Age at death | a | 14.5 | Age of onset of the disability | a | 11 | 13 |
| Constant from the age weighting function | β | 0.04 | Constant from the age weighting function | β | 0.04 | 0.04 |
| Standard expectation of life at age | L | 65.91 | Duration of disability | L | 2 | 1.5 |
|  | e | 2.72 |  | e | 2.72 | 2.72 |
|  |  |  | Disability weight | D | 0.12 | 0.51 |
| YLL |  | 36.93 | YLDs |  | 0.29 | 0.97 |
| DALYs saved (YLL + YLDs) |  | 38.20 |  |  |  |  |

***Girls age 14 years or less***

| **YLL** | | | **YLDs** | | | |
| --- | --- | --- | --- | --- | --- | --- |
| **Variables** | |  | **Variables** | | **Pre- AIDS** | **AIDS** |
| Age weighting modulation factor | K | 1 | Age weighting modulation factor | K | 1 | 1 |
| Age weighting constant | C | 0.1658 | Age weighting constant | C | 0.1658 | 0.1658 |
| Discount rate | r | 0.03 | Discount rate | r | 0.03 | 0.03 |
| Age at death | a | 14.5 | Age of onset of the disability | a | 11 | 13 |
| Constant from the age weighting function | β | 0.04 | Constant from the age weighting function | β | 0.04 | 0.04 |
| Standard expectation of life at age | L | 68.52 | Duration of disability | L | 2 | 1.5 |
|  | e | 2.72 |  | e | 2.72 | 2.72 |
|  |  |  | Disability weight | D | 0.12 | 0.51 |
| YLL |  | 37.11 | YLDs |  | 0.29 | 0.97 |
| DALYs saved (YLL + YLDs) |  | 38.38 |  |  |  |  |

***Women age 15-44 years***

| **YLL** | | | **YLDs** | | | |
| --- | --- | --- | --- | --- | --- | --- |
| **Variables** | |  | **Variables** | | **Pre- AIDS** | **AIDS** |
| Age weighting modulation factor | K | 1 | Age weighting modulation factor | K | 1 | 1 |
| Age weighting constant | C | 0.1658 | Age weighting constant | C | 0.1658 | 0.1658 |
| Discount rate | r | 0.03 | Discount rate | r | 0.03 | 0.03 |
| Age at death | a | 39.5 | Age of onset of the disability | a | 36 | 38 |
| Constant from the age weighting function | β | 0.04 | Constant from the age weighting function | β | 0.04 | 0.04 |
| Standard expectation of life at age | L | 44.01 | Duration of disability | L | 2 | 1.5 |
|  | e | 2.72 |  | e | 2.72 | 2.72 |
|  |  |  | Disability weight | D | 0.14 | 0.51 |
| YLL |  | 24.05 | YLDs |  | 0.37 | 1.01 |
| DALYs saved (YLL + YLDs) |  | 25.43 |  |  |  |  |

***Men age 15-44 years***

| **YLL** | | | **YLDs** | | | |
| --- | --- | --- | --- | --- | --- | --- |
| **Variables** | |  | **Variables** | | **Pre- AIDS** | **AIDS** |
| Age weighting modulation factor | K | 1 | Age weighting modulation factor | K | 1 | 1 |
| Age weighting constant | C | 0.1658 | Age weighting constant | C | 0.1658 | 0.1658 |
| Discount rate | r | 0.03 | Discount rate | r | 0.03 | 0.03 |
| Age at death | a | 39.5 | Age of onset of the disability | a | 36 | 38 |
| Constant from the age weighting function | β | 0.04 | Constant from the age weighting function | β | 0.04 | 0.04 |
| Standard expectation of life at age | L | 41.16 | Duration of disability | L | 2 | 1.5 |
|  | e | 2.72 |  | e | 2.72 | 2.72 |
|  |  |  | Disability weight | D | 0.14 | 0.51 |
| YLL |  | 23.64 | YLDs |  | 0.37 | 1.01 |
| DALYs saved (YLL + YLDs) |  | 25.02 |  |  |  |  |

***Men age 45 years and above***

| **YLL** | | | **YLDs** | | | |
| --- | --- | --- | --- | --- | --- | --- |
| **Variables** | |  | **Variables** | | **Pre- AIDS** | **AIDS** |
| Age weighting modulation factor | K | 1 | Age weighting modulation factor | K | 1 | 1 |
| Age weighting constant | C | 0.1658 | Age weighting constant | C | 0.1658 | 0.1658 |
| Discount rate | r | 0.03 | Discount rate | r | 0.03 | 0.03 |
| Age at death | a | 59.5 | Age of onset of the disability | a | 56 | 58 |
| Constant from the age weighting function | β | 0.04 | Constant from the age weighting function | β | 0.04 | 0.04 |
| Standard expectation of life at age | L | 22.26 | Duration of disability | L | 2 | 1.5 |
|  | e | 2.72 |  | e | 2.72 | 2.72 |
|  |  |  | Disability weight | D | 0.14 | 0.51 |
| YLL |  | 11.74 | YLDs |  | 0.26 | 0.69 |
| DALYs saved (YLL + YLDs) |  | 12.69 |  |  |  |  |

***Women age 45 years and above***

| **YLL** | | | **YLDs** | | | |
| --- | --- | --- | --- | --- | --- | --- |
| **Variables** | |  | **Variables** | | **Pre- AIDS** | **AIDS** |
| Age weighting modulation factor | K | 1 | Age weighting modulation factor | K | 1 | 1 |
| Age weighting constant | C | 0.1658 | Age weighting constant | C | 0.1658 | 0.1658 |
| Discount rate | r | 0.03 | Discount rate | r | 0.03 | 0.03 |
| Age at death | a | 59.5 | Age of onset of the disability | a | 56 | 58 |
| Constant from the age weighting function | β | 0.04 | Constant from the age weighting function | β | 0.04 | 0.04 |
| Standard expectation of life at age | L | 25.28 | Duration of disability | L | 2 | 1.5 |
|  | e | 2.72 |  | e | 2.72 | 2.72 |
|  |  |  | Disability weight | D | 0.14 | 0.51 |
| YLL |  | 12.48 | YLDs |  | 0.26 | 0.69 |
| DALYs saved (YLL + YLDs) |  | 13.42 |  |  |  |  |

DALYs saved (weighted proportional to the estimated use of donated blood by each group) = 25.4

**Calculation of DALYs saved per infection averted (without age-weighting)**

***Boys age 14 years or less***

| **YLL** | | | **YLDs** | | | |
| --- | --- | --- | --- | --- | --- | --- |
| **Variables** | |  | **Variables** | | **Pre- AIDS** | **AIDS** |
| Age weighting modulation factor | K | 0 | Age weighting modulation factor | K | 0 | 0 |
| Age weighting constant | C | NA | Age weighting constant | C | NA | NA |
| Discount rate | r | 0.03 | Discount rate | r | 0.03 | 0.03 |
| Age at death | a | 14.5 | Age of onset of the disability | a | 11 | 13 |
| Constant from the age weighting function | β | NA | Constant from the age weighting function | β | NA | NA |
| Standard expectation of life at age | L | 65.91 | Duration of disability | L | 2 | 1.5 |
|  | e | 2.72 |  | e | 2.72 | 2.72 |
|  |  |  | Disability weight | D | 0.12 | 0.51 |
| YLL |  | 28.72 | YLDs |  | 0.24 | 0.74 |
| DALYs saved (YLL + YLDs) |  | 29.70 |  |  |  |  |

***Girls 14 years or less***

| **YLL** | | | **YLDs** | | | |
| --- | --- | --- | --- | --- | --- | --- |
| **Variables** | |  | **Variables** | | **Pre- AIDS** | **AIDS** |
| Age weighting modulation factor | K | 0 | Age weighting modulation factor | K | 0 | 0 |
| Age weighting constant | C | NA | Age weighting constant | C | NA | NA |
| Discount rate | r | 0.03 | Discount rate | r | 0.03 | 0.03 |
| Age at death | a | 14.5 | Age of onset of the disability | a | 11 | 13 |
| Constant from the age weighting function | β | NA | Constant from the age weighting function | β | NA | NA |
| Standard expectation of life at age | L | 68.52 | Duration of disability | L | 2 | 1.5 |
|  | e | 2.72 |  | e | 2.72 | 2.72 |
|  |  |  | Disability weight | D | 0.12 | 0.51 |
| YLL |  | 29.07 | YLDs |  | 0.24 | 0.74 |
| DALYs saved (YLL + YLDs) |  | 30.05 |  |  |  |  |

***Women age 15-44 years***

| **YLL** | | | **YLDs** | | | |
| --- | --- | --- | --- | --- | --- | --- |
| **Variables** | |  | **Variables** | | **Pre- AIDS** | **AIDS** |
| Age weighting modulation factor | K | 0 | Age weighting modulation factor | K | 0 | 0 |
| Age weighting constant | C | NA | Age weighting constant | C | NA | NA |
| Discount rate | r | 0.03 | Discount rate | r | 0.03 | 0.03 |
| Age at death | a | 39.5 | Age of onset of the disability | a | 36 | 38 |
| Constant from the age weighting function | β | NA | Constant from the age weighting function | β | NA | NA |
| Standard expectation of life at age | L | 44.01 | Duration of disability | L | 2 | 1.5 |
|  | e | 2.72 |  | e | 2.72 | 2.72 |
|  |  |  | Disability weight | D | 0.14 | 0.51 |
| YLL |  | 24.43 | YLDs |  | 0.26 | 0.74 |
| DALYs saved (YLL + YLDs) |  | 25.44 |  |  |  |  |

***Men age 15-44 years***

| **YLL** | | | **YLDs** | | | |
| --- | --- | --- | --- | --- | --- | --- |
| **Variables** | |  | **Variables** | | **Pre- AIDS** | **AIDS** |
| Age weighting modulation factor | K | 0 | Age weighting modulation factor | K | 0 | 0 |
| Age weighting constant | C | NA | Age weighting constant | C | NA | NA |
| Discount rate | r | 0.03 | Discount rate | r | 0.03 | 0.03 |
| Age at death | a | 39.5 | Age of onset of the disability | a | 36 | 38 |
| Constant from the age weighting function | β | NA | Constant from the age weighting function | β | NA | NA |
| Standard expectation of life at age | L | 41.16 | Duration of disability | L | 2 | 1.5 |
|  | e | 2.72 |  | e | 2.72 | 2.72 |
|  |  |  | Disability weight | D | 0.136 | 0.505 |
| YLL |  | 23.64 | YLDs |  | 0.26 | 0.74 |
| DALYs saved (YLL + YLDs) |  | 24.64 |  |  |  |  |

***Men age 45 years and above***

| **YLL** | | | **YLDs** | | | |
| --- | --- | --- | --- | --- | --- | --- |
| **Variables** | |  | **Variables** | | **Pre- AIDS** | **AIDS** |
| Age weighting modulation factor | K | 0 | Age weighting modulation factor | K | 0 | 0 |
| Age weighting constant | C | NA | Age weighting constant | C | NA | NA |
| Discount rate | r | 0.03 | Discount rate | r | 0.03 | 0.03 |
| Age at death | a | 59.5 | Age of onset of the disability | a | 56 | 58 |
| Constant from the age weighting function | β | NA | Constant from the age weighting function | β | NA | NA |
| Standard expectation of life at age | L | 22.26 | Duration of disability | L | 2 | 1.5 |
|  | e | 2.72 |  | e | 2.72 | 2.72 |
|  |  |  | Disability weight | D | 0.14 | 0.51 |
| YLL |  | 16.24 | YLDs |  | 0.26 | 0.74 |
| DALYs saved (YLL + YLDs) |  | 17.24 |  |  |  |  |

***Women age 45 years and above***

| **YLL** | | | **YLDs** | | | |
| --- | --- | --- | --- | --- | --- | --- |
| **Variables** | |  | **Variables** | | **Pre- AIDS** | **AIDS** |
| Age weighting modulation factor | K | 0 | Age weighting modulation factor | K | 0 | 0 |
| Age weighting constant | C | NA | Age weighting constant | C | NA | NA |
| Discount rate | r | 0.03 | Discount rate | r | 0.03 | 0.03 |
| Age at death | a | 59.5 | Age of onset of the disability | a | 56 | 58 |
| Constant from the age weighting function | β | NA | Constant from the age weighting function | β | NA | NA |
| Standard expectation of life at age | L | 25.28 | Duration of disability | L | 2 | 1.5 |
|  | e | 2.72 |  | e | 2.72 | 2.72 |
|  |  |  | Disability weight | D | 0.14 | 0.51 |
| YLL |  | 17.72 | YLDs |  | 0.26 | 0.74 |
| DALYs saved (YLL + YLDs) |  | 18.73 |  |  |  |  |

NA means not applicable

DALYs saved (weighted proportional to the estimated use of donated blood by each group) = 24.5
